# Global report on preterm birth and stillbirth (3 of 7): evidence for effectiveness of interventions

**DOI:** 10.1186/1471-2393-10-S1-S3

**Published:** 2010-02-23

**Authors:** Fernando C Barros, Zulfiqar Ahmed Bhutta, Maneesh Batra, Thomas N Hansen, Cesar G Victora, Craig E Rubens, the GAPPS Review Group

**Affiliations:** 1 Post-Graduate Course in Health and Behaviour, Universidade Catolica de Pelotas, Brazil; 2 Division of Women & Child Health, Aga Khan University, Karachi 74800, Pakistan; 3 Divison of Neonatology, Department of Pediatrics, University of Washington School of Medicine, Seattle, Washington, USA; 4 Seattle Children's, Seattle, Washington, USA; 5 Universidade Federal de Pelotas, Pelotas 96001-970, Brazil; 6 Global Alliance to Prevent Prematurity and Stillbirth, an initiative of Seattle Children's, Seattle, Washington, USA; 7 Department of Pediatrics at University of Washington School of Medicine, Seattle, Washington, USA

## Abstract

**Introduction:**

Interventions directed toward mothers before and during pregnancy and childbirth may help reduce preterm births and stillbirths. Survival of preterm newborns may also be improved with interventions given during these times or soon after birth. This comprehensive review assesses existing interventions for low- and middle-income countries (LMICs).

**Methods:**

Approximately 2,000 intervention studies were systematically evaluated through December 31, 2008. They addressed preterm birth or low birth weight; stillbirth or perinatal mortality; and management of preterm newborns. Out of 82 identified interventions, 49 were relevant to LMICs and had reasonable amounts of evidence, and therefore selected for in-depth reviews. Each was classified and assessed by the quality of available evidence and its potential to treat or prevent preterm birth and stillbirth. Impacts on other maternal, fetal, newborn or child health outcomes were also considered. Assessments were based on an adaptation of the Grades of Recommendation Assessment, Development and Evaluation criteria.

**Results:**

Most interventions require additional research to improve the quality of evidence. Others had little evidence of benefit and should be discontinued. The following are supported by moderate- to high-quality evidence and strongly recommended for LMICs:

• Two interventions prevent preterm births—smoking cessation and progesterone

• Eight interventions prevent stillbirths—balanced protein energy supplementation, screening and treatment of syphilis, intermittant presumptive treatment for malaria during pregnancy, insecticide-treated mosquito nets, birth preparedness, emergency obstetric care, cesarean section for breech presentation, and elective induction for post-term delivery

• Eleven interventions improve survival of preterm newborns—prophylactic steroids in preterm labor, antibiotics for PROM, vitamin K supplementation at delivery, case management of neonatal sepsis and pneumonia, delayed cord clamping, room air (vs. 100% oxygen) for resuscitation, hospital-based kangaroo mother care, early breastfeeding, thermal care, and surfactant therapy and application of continued distending pressure to the lungs for respiratory distress syndrome

**Conclusion:**

The research paradigm for discovery science and intervention development must be balanced to address prevention as well as improve morbidity and mortality in all settings. This review also reveals significant gaps in current knowledge of interventions spanning the continuum of maternal and fetal outcomes, and the critical need to generate further high-quality evidence for promising interventions.

## Introduction

Global preterm birth and stillbirth rates may be improved with interventions directed toward women before and during pregnancy, labor and birth. Such interventions, along with those given to preterm newborns, may also improve preterm survival and other maternal, newborn and child health (MNCH) outcomes. Nearly 50 existing interventions are discussed in this article, and were selected on the basis of available evidence of impact, ability to be used in low- and middle-income countries (LMICs), and other factors discussed below. They are organized by stage and recipient:

Interventions directed toward the mother to prevent preterm birth or stillbirth

• Before pregnancy

• During pregnancy

• Pregnancy infections

• High-risk pregnancies

• Intrapartum interventions to prevent stillbirth

Interventions to improve preterm survival

• Intrapartum interventions given to the mother

• Postpartum interventions directed toward the preterm newborn

This article discusses the evidence base for these interventions, and gives special reference to LMICs. A recommendation is provided for each intervention. When available, the evidence is assessed for preterm birth, stillbirth, preterm survival and other MNCH outcomes.

This is the third article in a global report on preterm birth and stillbirth. The first two articles describe the definitions, data and known causes [1,2]. The next few articles discuss scaling up proven interventions, advocacy barriers and opportunities, and ethical considerations relating to these issues [3-5]. The concluding article presents recommendations for a Global Action Agenda [6].

## Methods

**Figure 1:**
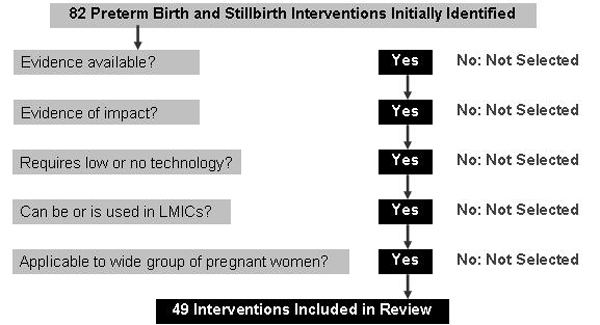
Intervention selection process.

This review systematically evaluates interventions to prevent preterm birth and stillbirth, and to improve survival among preterm newborns. More than 80 biologically plausible interventions were initially selected (Table 1). Figure 1 illustrates the final selection process for the 49 interventions included in this review.

**Table 1:** List of interventions

Selected	Interventions	Selected	Interventions
**Group 1:**	**Interventions given before pregnancy**	**Group 5:**	**Intrapartum interventions to prevent stillbirth**
X	Birth spacing	X	Birth preparedness
X	Periconceptional folate	X	Use of partogram
X	Indoor air pollution control	X	Fetal movement monitoring
	Prevention of female genital mutilation	X	Emergency obstetric care
		X	Cesarean section for breech presentation
**Group 2:**	**Interventions given during pregnancy**	X	Elective induction of labor for post-term delivery
X	Smoking cessation programs	X	Elective induction of term PROM
X	Balanced protein energy supplementation	X	Home delivery vs. facility delivery
X	Multiple micronutrient supplementation		Instrumental deliveries (forceps versus vacuum)
X	Iron and folate supplementation		Amnioinfusion
X	Zinc supplementation		Cervical ripening and induction of labor with diff erent
X	Magnesium sulfate supplementation		prostaglandins
X	Calcium supplementation		COX inhibitors for preterm labor
X	Supplementation with long-chain polyunsaturated fatty acids		Magnesium sulphate for treatment of preeclampsia/eclampsia or
X	Cardiotocographic monitoring		preterm labor
X	Doppler and late ultrasound monitoring		Maternal hyperoxygenation
	Anti-platelet agents in pregnancy, including aspirin		
	Anti-malarials	**Group 6:**	**Antepartum and intrapartum interventions to improve preterm survival**
	Anti-oxidants	X	Prophylactic corticosteroid therapy in preterm labor
	Vitamin A/Beta-Carotene supplementation	X	Antibiotics for PROM/PPROM
		X	Antibiotics for preterm labor with intact membranes
**Group 3:**	**Interventions for pregnancy infections**	X	Delayed cord clamping
X	Screening and treatment of syphilis		Vitamin A to the mother
X	Intermittent presumptive treatment during pregnancy (IPTp) for malaria		
X	Insecticide-treated mosquito nets (ITNs)	**Group 7:**	**Postnatal interventions to improve preterm survival**
X	Screening and treatment of asymptomatic bacteriuria	X	Neonatal resuscitation
X	Screening and treatment of bacterial vaginosis	X	Vitamin A supplementation
X	Prevention of mother-to-child transmission of HIV	X	Vitamin K supplementation
X	Anti-helminthic treatment	X	Zinc supplementation
X	Screening and treatment of periodontal disease	X	Selenium supplementation
		X	Chlorhexidine treatment on the cord
**Group 4:**	**Interventions for pregnancies with high-risks of PTB or SB**	X	Case management of neonatal sepsis and pneumonia
X	Progesterone	X	Kangaroo mother care (KMC)
X	Cervical cerclage	X	Early breastfeeding
X	Multivitamins for HIV+ women	X	Thermal care
	Amniotic fluid volume assessment	X	Application of continued distending pressure or CPAP to the
	Antepartum fetal heart rate monitoring with cardiotocography		lungs for RDS
	Cervical pessaries to prevent preterm birth	X	Intravenous immune globulin (IVIG)
	Fetal biophysical test scoring	X	Surfactant therapy for RDS
	Home versus hospital monitoring for high-risk pregnancies		Emollient therapy
	In-hospital fetal surveillance unit		Hand washing
	Intrapartum cardiotocography and pulse oximetry		Prophylaxis of eye infection
	Management of gestational diabetes mellitus		Use of Appropriate Low-cost Technology (incubators, techniques
	Non stress testing or vibroacoustic stimulation		for minimally invasive intravenous access, protection against the
	Use of the partograph		excessive use of oxygen)
	Fetal movement monitoring		
	Heparin in pregnancy		
	Management of intrahepatic cholestasis		
	Pelvimetry		
	Plasma exchange		
	Pregnancy risk screening		
	Ultrasound scanning		

X indicates interventions described in the text. Interventions without the X were reviewed but not included due to one or more of the following reasons: (a) the available evidence was very limited; (b) there was no evidence of an impact; (c) the intervention requires high technology; (d) the intervention is seldom used; (e) the intervention was applicable to a small subgroup of pregnant women.

Approximately 2,000 studies were reviewed on interventions addressing preterm birth (or low birth weight), stillbirth (or perinatal mortality), and preterm survival. This number includes systematic reviews and meta-analyses, along with stand-alone papers from observational or experimental studies. Interventions with a beneficial impact on other maternal, newborn and child health outcomes, but that did not influence preterm birth or stillbirth outcomes, were not considered.

The search engines used were PubMed and The Cochrane Library. Search terms included "stillbirth*," "perinatal mortality," "fetal death*," "preterm*," "low birth weight"and "low birthweight." Targeted search terms were also used for each intervention (e.g., "iron" AND "supplementation" AND "pregnancy"; and "cervical cerclage" AND "pregnancy"), with humans as limits. These terms are defined in article 1 of this global report [1].

The last search date was December 31, 2008. (Note that papers published after this date may influence key findings in this article.) The emphasis was to identify randomized and quasi-randomized study designs. Where such trials were not available, observational studies were included. When a high-quality meta-analysis was available, it served as the basis for the review, and was updated with results of more recent studies when available.

Perinatal mortality studies are also considered, as some did not separate stillbirths from overall perinatal mortality. Few studies distinguished between antepartum and intrapartum stillbirths. Studies reporting on overall low birth weight rates, or mean birth weights, are also included when preterm birth was not reported as a separate outcome.

For each intervention, an adaptation of the Grades of Recommendation Assessment, Development and Evaluation (GRADE) criteria [7,8] was used to rate the level of available evidence into either high, moderate, low or very low (see Table 2).

**Table 2:** GRADE Criteria for Quality of Evidence

Quality of Evidence	Study Design	Interpretation
High	Randomized Controlled Trials (RCTs)	Further research is very unlikely to change our confidence in the estimate of effect.
Moderate	Low-Quality Randomized Trials or High-Quality Observational Studies	Further research is likely to have an important impact on our confidence in the estimate of effect and may change the estimate.
Low	Observational Studies	Further research is very likely to have an important impact on our confidence in the estimate of effect and is likely to change the estimate.
Very Low	Any Other Evidence	Any estimate of effect is very uncertain.

The GRADE system starts from a pre-established quality of evidence rating for each type of study design, but allows flexibility for lowering or increasing this rating. For example, results from randomized controlled trials (RCTs) may be downgraded from "high" to "moderate" quality if there are methodological shortcomings such as lack of precision, the possibility of reporting bias or other limitations to study quality. On the other hand, results from observational studies may be upgraded from "low" to "moderate" if the studies are particularly strong (e.g., when confounding variables are unlikely or when there is a dose-response gradient).

The original GRADE system requires individual ratings for each study included in the review. The overall rating provided in this review is based on the collective appraisal of all publications on each intervention, since it was largely based on existing meta-analyses or on fresh pooled analyses carried out for stillbirth interventions and some preterm postnatal interventions.

A major advantage of the GRADE system, compared to other classifications, is it provides a practical recommendation regarding each intervention, assessing the extent to which one can be confident adherence to a recommendation will do more good than harm. The recommendation is based on the quality of evidence, how the evidence may be translated to practice in a specific setting such as LMICs, the level of baseline risk, and on potential tradeoffs between expected benefits, harms and costs.

The recommendation for each intervention was then placed in one of the following categories:

• Strong in favor

• Weak in favor

• Weak against

• Strong against

Recommendations against the intervention does not necess arily indicate there is evidence the intervention does harm; such recommendations most often reflect no evidence of benefit, and due to the costs incurred there are no reasons for further implementation.

In these assessments, recommendations are provided not only for the main outcomes in this review, but also for the intervention's impact on other MNCH outcomes when relevant. For many interventions, the recommendations for preventing stillbirth or preterm birth conflict with the recommendation for overall MNCH outcomes. This will be further discussed in the next article on the delivery of interventions.

## Results

The 49 interventions reviewed below are classified according to service delivery strategies across the continuum of care. Table 1 shows all interventions considered in this review. The narrative below is restricted to interventions for which a reasonable amount of evidence is present, and, or, those relevant to low- and middle-income countries. This review of the evidence indicates a remarkable paucity of data on preterm birth and stillbirth for many potentially relevant interventions.

Table 3 summarizes the assessment of the quality of evidence and level of recommendation for each intervention using the GRADE system. These recommendations consider the preterm birth and stillbirth outcomes, as well as other important maternal and neonatal outcomes. It is important to note that most studies reviewed lacked statistical power to pick up as statistically significant an effect on stillbirth, as their main outcomes were more frequently pregnancy and childbirth-related conditions. In addition, many studies—particularly from LMICs—did not report on preterm birth as an outcome.

**Table 3:** Summary of assessments for preterm birth and stillbirth interventions (based on GRADE system)

		Quality of evidence				Recommendation for Implementation			
		
Intervention (by stage or recipient)	SB	PMR	PTB	LBW	Other MNCH Outcomes	SB/PMR	PTB	LBW	Other MNCH Outcomes
**Interventions given before pregnancy**								
Birth Spacing	Low	Mod	Mod*	Mod*	Mod*	Weak	Weak	Weak	Strong: Maternal and Child Mortality
Periconceptional Folate	Mod	Mod	Low	Low	High	Weak Against	Weak	Weak	Strong: Neural Tube Defects
Indoor Air Pollution Control	Low		Very Low**	Low	High	Weak	Weak Against	Weak	Strong: Respiratory Infections
**Interventions given during pregnancy**								
Smoking Cessation Programs	Low	Low	High	High	High	Weak	Strong	Strong	Strong: Smoking-Related Diseases
Balanced Protein Energy Supplementation	High	-	HighNo Effect	High	-	Strong	Strong Against	Strong	Strong: Infant Mortality
Multiple Micronutrient Supplementation	High No Effect	Mod	HighNo Effect	High	High (Maternal Anemia, I MR)	Weak Against	Strong Against	Strong	Weak Against: Neonatal Mortality
Iron and Folate Supplementation	Mod No Effect**	Mod No Effect**	Mod No Effect**	Mod No Effect**	High (Maternal Anemia)	Weak Against	Weak Against	Weak Against	Strong: Anemia
Zinc Supplementation	HighNo Effect	HighNo Effect	High High	HighNo Effect	Mod (Reduced C-Sections)	Strong Against	Weak	Strong Against	Weak
Magnesium Sulfate Supplementation	High	High	Mod**	Mod**	High(Cerebral Palsy)	Strong Against	Weak Against	Weak Against	Weak: Supplementation Strong: Treatment of Eclampsia and Prevention of Cerebral Palsy
Calcium Supplementation	HighNo Effect	-	Mod	Mod	High (Preeclampsia)	Strong Against	Weak	Weak	Strong: Preeclampsia
Supplementation with Fatty Acids	-	-	HighNo Effect	HighNo Effect	-	-	Strong Against	Strong Against	-
Cardiotocographic Monitoring	Low	Mod	-			Weak			
Doppler and Late Ultrasound Monitoring	HighNo Effect		-	-		Strong Against	-	-	
**Pregnancy infections**								
Screening and Treatment of Syphilis	Mod*	Very Low**	Mod*	Mod*	Mod*	Strong	Weak	Weak	Strong: Congenital Syphilis
Intermittent Presumptive Treatment During Pregnancy (IPTp) for malaria	Mod	High	Low**	High	High	Strong	Weak	Strong	Strong: Maternal Malaria
Insecticide-Treated Mosquito Nets (ITNs)	High	-	Low**	High	High	Strong	Weak Against	Strong	Strong: Maternal Malaria
Screening and Treatment of Asymptomatic Bacteriuria	**	**	Low**	High	High (Maternal Morbidity)		Weak	Strong	Strong: Pyelonephritis, Maternal Morbidity
Screening and Treatment of Bacterial Vaginosis	-	-	HighNo Effect	HighNo Effect	-	-	Strong Against	Strong Against	-
Prevention of Mother-to- Child Transmission of HIV	Low	Low	Low		High (PMTCT)	**	**	**	Strong: PMTCT
Anti-Helminthic Treatment	Low	Low	Low	Low	High (Maternal Hookworm)	Weak Against	Weak Against	Weak Against	Strong: Maternal Anemia
Screening and Treatment of Periodontal Disease		-	Mod**	Mod**	Low (Maternal Oral Health)		Weak Against	Weak Against	Weak: Maternal Oral Health
**Interventions for pregnancies with high-risks of preterm birth or stillbirth**								
Progesterone	Mod		High	High	-	Weak Against	Strong	Strong	-
Cervical Cerclage	HighNo Effect		High No Effect		-	Weak Against	Strong Against	-	-
Multivitamins for HIV+ Women	Low	Low	Mod No Effect	Mod No Effect	-	Weak Against	Weak Against	Weak Against	-
**Intrapartum interventions to prevent stillbirths**								
Birth Preparedness	High	Mod	-	-		Strong	-	-	
Use of Partogram	Low	Low	-	-		Weak	-	-	
Fetal Movement Monitoring	Low	- -	-	-		Strong Against	-	-	
Emergency Obstetric Care	Mod*	Mod*	-		Mod (Maternal Outcomes)	Strong	-	-	Strong: Maternal Mortality
Cesarean Section for Breech Presentation	-	High	-	-	-	Strong	-	-	
Elective Induction for Post-Term Delivery	Mod	High	-	-		Strong	-	-	
Elective Induction for Term PROM		Mod			High (Maternal and Infant Outcomes)	Weak			Strong: Maternal and Infant Outcomes
Home Delivery		Mod	-	-		Weak Against			
**Intrapartum interventions to improve preterm survival**								
Prophylactic Steroids in Preterm Labor	-	-	High	-	High(Neonatal Morbidity)	-	Strong	-	Strong: Neonatal Morbidity
Antibiotics for PROM	-	-	High	-	High(Neonatal Morbidity)		Strong		Strong: Neonatal Morbidity
Antibiotics for Preterm Labor with Intact Membranes	-	-	High	-			Strong Against		
Delayed cord clamping			High				Strong		
**Postnatal interventions to improve preterm survival**								
Neonatal Resuscitation									
• Modes of oxygen delivery and resuscitation techniques: CPR			Low		-		-		-
• Modes of delivery and techniques: bag and mask			Mod		-		-		-
• CPR techniques: long- & short-term outcomes			Low		-		-		-
• Room air (vs. 100% oxygen) for resuscitation			High		-		Strong		-
Training programs for health facilities			Mod		-		Weak		-
Vitamin A supplementation			Mod		-		Weak Against		-
Vitamin K supplementation			Mod		Mod (antenatal)		Strong		Weak: antenatal
Zinc supplementation			Low		-		Weak Against		-
Selenium supplementation			Mod		-		Weak		-
Chlorhexidine treatment on the cord			Mod		-		-		-
Case management of neonatal sepsis and pneumonia			Mod		-		Strong		-
Kangaroo mother care (KMC)									
Hospital-based KMC			High		-		Strong		-
Community-based KMC			Low		-		-		-
Early breastfeeding			High		Mod (neonatal mortality)		Strong		-
Thermal care (skin to skin; plastic wraps)			Mod		-		Strong		-
Application of continued distending pressure to the lungs for RDS			High		High (reducing chronic lung disease, and as alternative to intubation) Low pneumothorax		Strong		Strong: bronchopulmonary dysplasia
Intravenous immune globulin (IVIG)			High				Weak		
Surfactant therapyfor RDS			High		High (reducing chronic lung disease)		Strong		Strong

(*) Due to ethical issues, RCTs are not possible.

(**) Limited evidence, or outcomes not reported.

(-) Not applicable or no evidence

### Interventions given before pregnancy

#### Birth spacing

A number of hypotheses explain why short or long birth intervals could affect perinatal health. For short intervals, maternal nutrition depletion is the most often proposed; women do not have time to recover from the expenses related to pregnancy and lactation before getting pregnant again. For long intervals, the reasons are less clear, but it appears reproductive capacity declines after delivery, and that diseases affecting the mother could have an effect on both fertility and the health of the baby [9]. Optimally-spaced births can potentially reduce fetal and maternal morbidity and mortality.

Access to modern family planning methods is still problematic in many low-income countries [10]. It is calculated that birth spacing promotion in countries with high birth rates could reduce one third of maternal deaths and 10% of childhood mortality [11]. After a birth, the interval before attempting a new pregnancy should be at least 24 months to reduce the risk of adverse maternal and infant outcomes [12].

##### Preterm birth

A meta-analysis of observational studies [9] showed a 40% increase in preterm births when the birth interval was shorter than 6 months, relative to babies born after a birth interval of 18-23 months (RR 1.40; CI 1.24-1.58). The corresponding odds ratios were 1.61 (95% CI 1.391.86) for low birth weight and 1.26 (95% CI 1.18-1.33) for IUGR. A 20% increase in preterm births was also found when the birth interval was 60 months or longer (OR 1.20; 95% CI 1.17-1.24). There are no intervention studies, however, showing that promoting birth spacing will reduce either preterm births or low birth weight.

##### Stillbirth

Stephansson et al. [13], using a logistic regression analysis on the Swedish registry data, showed that compared with interpregnancy intervals (IPIs) 12-25 months, very short IPIs (0-3 months) were linked to an increased risk of stillbirth, though nonsignificant (adjusted OR = 1.3; 95% CI: 0.8-2.1). IPIs greater than 72 months also increased women's risk of stillbirth in this study (adjusted OR = 1.5; 95% CI: 1.1-2.1). Other studies have also shown a link between increased risk of fetal loss and short or long IPIs [14,15].

##### Assessment

The quality of evidence relating birth spacing to preterm birth and low birth weight is moderate, as it is based on a large number of carefully conducted observational studies. While it is not expected that randomized trials will be conducted on the value of birth spacing in the future, there is a critical need for evaluating the effectiveness of various strategies to promote birth spacing, induced terminations and prevention of unwanted pregnancies [16]. The level of recommendation is, therefore, weak in favor of this intervention to prevent preterm birth and low birth weight.

The overall quality of evidence of the impact of birth spacing on perinatal mortality is moderate, but data on stillbirths are sparse. Therefore, this intervention has low evidence of benefit for preventing stillbirth and the recommendation is weak in favor of its use for this outcome. Further evaluation of interventions promoting birth spacing is needed with reference to stillbirth outcomes. However, birth spacing is strongly recommended due to its important effects on both maternal and child health outcomes [12].

#### Periconceptional folate

Folic acid is a key element involved in DNA metabolism and necessary for red cell formation and correct closure of the neural tube. Almost all people who do not consume supplemental folic acid are folate deficient, and periconceptional folate supplementation reduces neural tube defects by 50-70% [17]. It is calculated that a 90% coverage of folate use in low- and middle-income countries would reduce nearly 80% of such defects [18].

##### Preterm birth

No intervention studies show an effect of periconceptional folate supplementation on preterm birth or low birth weight. Three observational studies conducted in the United States were identified in this review. The first found a two-fold increased risk of preterm birth and low birth weight in women with low daily folate intake and low serum folate levels at 28 weeks of gestation [19]. Another observational study, conducted before and after folate fortification programs in California, found small reductions in adjusted risks after the fortification for low birth weight (RR 0.94; 0.93-0.96) and preterm birth (RR 0.96; 0.94-0.97) [20]. A third observational study [21] showed that preconceptional folate supplementation for longer than one year was associated with a reduced incidence of spontaneous preterm birth between 28 and 32 weeks (hazard ratio 0.45, 0.23-0.85), but no significant effect was noted between 32-36 weeks of gestation.

##### Stillbirth

A Cochrane review, including three trials that reported perinatal outcomes, concluded that periconceptional folic acid supplementation is of proven benefit in reducing neural tube defects, reflected by a statistically significant reduction in its rate (RR 0.28; 95% CI 0.13-0.58), but impact on stillbirths is that of a 22%, nonsignificant reduction (RR 0.78; 95% CI 0.34-1.78) [22].

##### Assessment

The quality of evidence is low for preterm birth, but because the three observational studies point in the same direction, the recommendation is weak in favor of the intervention. The quality of evidence is moderate for stillbirths given the wide uncertainty in the results; the intervention cannot be recommended exclusively for preventi ng stillbirth and thus received a weak recommendation against the intervention for this outcome. Nevertheless, periconceptional folate is strongly recommended for other MNCH outcomes due to its protective effect on neural tube defects. Further studies are needed to assess potential long-term benefits of folic acid supplementation during pregnancy.

#### Indoor air pollution

The daily energy needs of around 50% of the world's popul ation are met by the burning of solid fuels [23]. Cooking is usually the domain of women, especially in developing countries even during pregnancy. Their homes are usually crowded and poorly ventilated, leading to intense smoke exposure that could harm the growing fetus as smoke metabolites cross the placental barrier.

##### Preterm birth

Observational studies in Guatemala, Pakistan and Zimbabwe show an association between this practice and decreased birth weight, but no information is available on preterm births [24,25]. In addition, no intervention studies are available on the impact of reduced indoor air pollution on either low birth weight or preterm birth.

##### Stillbirth

Studies on the impact of indoor air pollution on stillbirths are restricted to South Asian countries. Mavalankar, in India, found a nonsignificant increase in stillbirth risk in a case control study among women exposed to smoke (OR 1.5; 95% CI 1.0-2.1) [26]. Another population-based Indian study showed that women using biomass fuels had a significantly higher risk of stillbirth than those utilizing cleaner fuels (OR 1.44; 95% CI 1.04-1.97) [27]. Siddiqui et al. from Pakistan reported nearly a two-fold greater risk of stillbirths in pregnant women exposed to biomass fuel (OR 1.90; 95% CI 1.10-3.20) [28].

##### Assessment

There is a lack of studies relating indoor air pollution to preterm birth, especially randomized controlled trials [29,30]. Therefore the quality of evidence is very low and the recommendation is weak against the intervention for this particular outcome. For low birth weight and stillbirths, the quality of the evidence is low, as it is largely based on observational studies. However, the intervention is strongly recommended for the prevention of respiratory infections [31,32]. Future studies of measures to reduce indoor air pollution must also measure pregnancy and neonatal outcomes.

### Interventions given during pregnancy

#### Smoking cessation programs

More than 80% of all smokers now reside in low- and middle-income countries, with an estimated prevalence of 49% among men and 8% among women [33,34]. Recent prevalence studies of smoking during pregnancy also show wide variability, [35] with rates above 25% in South America [36], 8% in urban Africa [37] and 18% in the Pacific Islands [38].

Mechanisms that increase the risks of preterm birth and stillbirth among smokers are not clear. It is known, however, that both nicotine and carbon monoxide are potent vasoconstrictors, produce placental damage, and decrease the uteroplacental blood flow [39].

##### Preterm birth

Cigarette smoking is a well-known cause of preterm birth and intrauterine growth restriction [40]. In a Cochrane review published in 2004, smoking cessation interventions significantly reduced low birth weight (RR 0.81; 0.70-0.94) and preterm birth (RR 0.84; 0.72-0.98), as well as smoking during pregnancy [41]. It should be noted, however, that the experience of smoking cessation programs is predominantly from high-income countries [42].

##### Stillbirth

Notwithstanding the established benefits to mother and fetus, there are few studies that have reported the effect of smoking cessation on perinatal outcomes and stillbirths. One cohort study from Sweden shows increased risk of stillbirths among smokers [43].

##### Assessment

The quality of evidence is high for both preterm birth and low birth weight, and the intervention has a strong recommendation, especially in countries where smoking has a high prevalence during pregnancy. The strong recommendation is reinforced by other well-known effects of smoking on health. The lack of studies on smoking cessation and stillbirth outcomes points to a data gap that should be filled. Maternal exposure to second-hand smoke is an additional research gap. It should also be noted that all studies reviewed originate from high-income settings, and therefore intervention studies in low- and middle-income settings are needed.

#### Balanced protein energy supplementation

Maternal undernutrition is still a major problem in the world's poor countries [44]. Energy intake in pregnancy is positively associated with fetal growth [40]. In populations with food insecurity and high rates of maternal undernutrition, balanced energy protein supplementation—including up to 25% of the total energy content in the form of protein—may improve fetal growth and reduce the risk of fetal and neonatal death.

##### Preterm birth

A Cochrane review of seven trials of balanced protein energy supplementation for pregnant women did not show a significant effect on preterm births (RR 0.83; 0.65-1.06), but intrauterine growth restriction was reduced by 32% (RR 0.68; 0.54-0.86) [45]. Five of the seven trials were carried out in LMICs.

##### Stillbirth

The same review showed a significant reduction in the risks of stillbirth (RR 0.55; 95% CI 0.31-0.97) and a near-significant reduction in neonatal deaths (RR 0.62; 95% CI 0.37-1.05) [45].

##### Assessment

The quality of evidence is high and the intervention is not recommended for preventing preterm birth. However, this intervention is strongly recommended in appropriate populations (food insecure households and mothers with low body mass index) due to its affect on fetal growth and on preventing stillbirth.

#### Multiple micronutrient supplementation

Low serum levels of micronutrients such as iron, folate and zinc, are highly prevalent among pregnant women in low-income settings, and are associated with preterm birth and stillbirth [44,46-48]. Undernourished pregnant women consume less vitamins and minerals in their diets and have reduced blood volume and decreased uterine blood flow [49]. As a consequence, different nutritional interventions have been tried for women at high risk of nutritional deficiencies, including supplements of specific micronutrients, such as zinc, iron and folate, magnesium and calcium, given either singly or in combination.

##### Preterm birth

A review of nine RCTs comparing multiple micronutrient supplementation during pregnancy with either no supplements, two or fewer micronutrients, or placebo, showed a reduction in low birth weight (RR 0.84; 95% CI 0.74-0.95), IUGR (RR 0.92; 95% CI 0.86-0.99) and maternal anemia (RR 0.61; 0.52-0.71), but no effect on preterm birth [50]. A more recent trial in Tanzania compared multivitamins with placebo; the intervention group showed a reduction in low birth weight (RR 0.82; 95% CI 0.70-0.95) and in the frequency of small for gestational age newborns (RR 0.77; 95% CI 0.68-0.87), but no effect on preterm births or fetal mortality [51].

A 2006 meta-analyses of multiple micronutrient supplementation compared with iron and folic acid found no differences in maternal or neonatal outcomes [50]. However, when a 2008 RCT from Indonesia [52] was added to this meta-analysis, a significant reduction in low birth weight was observed (OR 0.84; 95% CI 0.74-0.95) [53].

##### Stillbirth

A reasonably large number of studies have recently evaluated the impact of multiple micronutrients on perinatal mortality and stillbirth outcomes. A recent meta-analysis was conducted, including trials published since the most recent Cochrane reviews, of the impact of multiple micronutrient supplementation in pregnancy [54]. The meta-analysis (nine RCTs, N=40,222 women, N=20,277 intervention group, N=19,945 controls) compared the impact on stillbirths of multiple micronutrient supplementation during pregnancy (intervention) with either iron or iron and folate (controls) and found a nonsignificant trend toward reduced stillbirths among the intervention group versus the control group (RR 0.91, 95% CI: 0.80-1.03).

One note of caution was provided by the report of increased birth asphyxia among babies in the intervention group in two micronutrient supplementation RCTs carried out in Nepal [55-57]. Their pooled analysis showed increased rates of perinatal and neonatal mortality (RR 1.52; 95% CI 1.03-2.25) in the intervention group. However, such an effect was not found in the Indonesia randomized trial, where infant mortality was 18% lower among infants whose mothers were supplemented with micronutrients [52]. In sites where these RCTs were carried out, most births took place either at home or in health posts, with limited access to emergency obstetric care. In Indonesia however, the study area had trained midwives and a signifcnatly greater proportion of births were assisted by skilled birth attendants. Thus this intervention must be viewed in the context of health system functionality and might provide benefits in circumstances where skilled care and facility births are available [58].

##### Assessment

There is high quality evidence the intervention has a significant impact on low birth weight but no effect on preterm births or stillbirths. However, due to moderate-quality evidence of increased neonatal mortality in South Asia, more information is necessary from effectiveness trials in different health systems settings before the intervention can be recommended for scaling up.

#### Iron and folate supplementation

Iron and folate are key elements for red cell production and iron-deficiency anemia, the most common nutrient deficiency among pregnant women. Routine iron or iron-folate supplementation is recommended for correcting anemia during pregnancy [59].

##### Preterm birth

A meta-analysis of four trials of iron supplementation during pregnancy, with and without folic acid, [60] showed a reduction in anemia but no significant effect on preterm birth (RR 0.76; 95% CI 0.47-1.24) or low birth weight (RR 0.59; 95% CI 0.23-1.49).

##### Stillbirth

The impact of iron or iron-folate supplementation on the prevention of stillbirth is mixed, [54] partly because most studies are underpowered to detect differences. The Cochrane review by Pena-Rosas et al. on antenatal iron supplementation found a nonsignificant risk of increased perinatal mortality with iron supplementation compared to placebo (RR 2.50, 95% CI 0.10-59.88) [60]. Another study from The Gambia, reported stillbirth rates of 2.9% vs. 4.3% in intervention (200 mg oral ferrous sulphate) and control groups, respectively [61].

##### Assessment

There is moderate evidence of a lack of effect for iron-folate supplementation on preterm birth, low birthweight and stillbirth. Although this is based on randomized trials, the quality of the evidence is moderate, because the confidence intervals are very wide. The recommendation—based exclusively on these outcomes—is weak against supplementation. Nevertheless, the well known impact of iron and folate in the prevention of maternal anemia result in a strong recommendation in favor of supplementation.

#### Zinc supplementation

Zinc plays an important role in many biological functions, including protein synthesis and nucleic acid metabolism [62]. Mild to moderate zinc deficiency is common in low-income settings, where pregnant women tend to consume less than the recommended daily intake of 15 mg [63].

##### Preterm birth

In a Cochrane review of 13 trials, seven of which were from low-middle income countries, zinc supplementation during pregnancy led to a modest reduction of 14% (95% CI 0.76-0.98) in preterm birth, but had no effect on low birth weight (RR 1.05, 95% CI 0.94-1.17), suggesting this effect may be restricted to large preterm babies [64].

##### Stillbirth

The recent Cochrane review of 9000 pregnancies mentioned above had 7 studies that reported no effects on stillbirth and perinatal mortality outcomes. The single study [55] undertaken in Nepal, a population with low maternal zinc status, reported no effect on stillbirths or other perinatal deaths (RR 1.03, 95% CI 0.71-1.51). A recent review of the subject [65] also did not report any stillbirth or other perinatal outcomes [55].

##### Assessment

The quality of evidence for zinc supplementation on stillbirth, preterm birth and low birth weight is high. The intervention is not recommended for the prevention of low birth weight or stillbirths, and has a weak recommendation for preventing preterm births, given its small effect size. No firm conclusion was reached on the effect of maternal zinc supplementation on stillbirth.

#### Magnesium sulfate supplementation

Magnesium sulfate reduces uterine contractility both in vivo and in vitro, indicating this intervention may prevent preterm birth and stillbirth [66].

##### Preterm birth

A meta-analysis of seven intervention trials with oral magnesium treatment starting before the 25th week of gestation showed a lower frequency of preterm birth (RR 0.73, 95% CI 0.57-0.94), as well as a reduction in low birth weight (RR 0.67, 95% CI 0.46 to 0.96) and improved maternal outcomes (hemorrhage and hospital admissions). However, authors of the Cochrane review noted that the studies included in the review had important shortcomings [67].

##### Stillbirth

The Cochrane review [67] included three RCTs with 1954 recipients that reported stillbirth outcomes and showed no impact (RR 1.0, 95% CI 0.29-3.44).

##### Assessment

The quality of evidence is moderate for both preterm birth and low birth weight, and there is a weak recommendation against the intervention until better studies become available. There is fairly consistent evidence that magnesium has no impact on stillbirth, and the recommendation is strong against the intervention for this outcome. A recent Cochrane review shows a reduced risk of cerebral palsy in the offspring of high-risk women who received magnesium sulfate [68] and thus this intervention should be evaluated further with a range of alternative outcomes.

#### Calcium supplementation

It is estimated that hypertension complicates 5% of all pregnancies and 11% of first pregnancies, accounting for nearly 40,000 maternal deaths annually [69]. Due to the known inverse association between calcium intake during pregnancy and hypertension, [70], supplementation has been tried in a number of studies.

##### Preterm birth

Calcium supplementation trials in low-risk women in populations with low-calcium diets reduced the risk of preeclampsia (RR 0.48; 95% CI 0.33-0.69) but its impact on preterm birth (10 trials, RR 0.81; 95% CI 0.64-1.03) and low birth weight (8 trials, RR 0.84, 95% CI 0.68-1.03) was not quite significant [71]. However, when the analysis was restricted to the four small studies including 568 women at high risk of preeclampsia, there was a significant decrease in preterm birth (RR 0.45, 95% CI 0.24 to 0.83).

In a recent small placebo-controlled RCT in low-risk Indian primigravidae with low dietary calcium, calcium supplementation led to less preeclampsia (OR 0.31; 95% CI 0.15-0.63) and significantly fewer preterm births (OR 0.51; 95% CI 0.28-0.93) [72].

##### Stillbirth

The Cochrane review [71] reported stillbirth and neonatal death before discharge in 10 trials with 15,141 participants, but showed no impact (RR 0.89, 95% CI 0.79-1.09).

##### Assessment

The quality of evidence for preterm birth and low birth weight is moderate, and the intervention has a weak recommendation for preventing preterm birth. For stillbirth, the quality of evidence is high suggesting a lack of effect. Therefore, the intervention is not recommended for these particular outcomes. However, it is strongly recommended for preventing preeclampsia in populations with low levels of dietary intake of calcium.

#### Supplementation with long-chain polyunsaturated fatty acids

Long-chain polyunsaturated fatty acids (LCPUFA) are precursors to the 3-series prostaglandins, which modulate inflammatory and vascular effects [73]. Because hypertension during pregnancy and preeclampsia are associated with vasoconstriction and endothelial damage, it is postulated these fatty acids play a beneficial role.

##### Preterm birth

Five randomized trials found that marine oil supplementation to low-risk women did not show any effect on preterm births (RR 0.92, 95% CI 0.79, 1.07), low birth weight, or the risk of preeclampsia [74]. Two RCTs showed LCPUFA supplementation was associated with a significantly lower rate of early preterm birth (<34 weeks of gestation) (RR 0.39, 95 % CI 0.18, 0.84) [75].

##### Stillbirth

No reported outcomes.

##### Assessment

The quality of evidence is high showing no effect of the intervention to prevent preterm birth and low birth weight. The recommendation against the intervention is strong for these outcomes given the current state of evidence. Further studies are needed on a possible effect on early preterm birth.

#### Cardiotocographic monitoring

One Cochrane review and two observational studies testing the impact of antepartum cardiotocography (including both NST and CST) on perinatal outcomes were identified [76-78]. The Cochrane review by Pattison & McCowan [76] included four studies (N=1,588 pregnancies) of the impact of cardiotocography use on perinatal mortality in high- or intermediate-risk pregnancies. The trial reported a trend toward increased perinatal mortality in the cardiotocography group versus controls receiving no monitoring or whose cardiotocography results were concealed from the clinician; (3 trials, N=1279 pregnancies, OR 2.85, 95% CI: 0.99-7.12). However, the studies were underpowered to assess such an impact. The observational studies do indicate a correlation between non-reassuring cardiotocographic traces and adverse perinatal outcomes, including stillbirth. While there are few RCTs evaluating the impact of cardiotocographic monitoring on reducing stillbirth, apparent reductions in stillbirth rates have followed the incorporation of the stress testing and cardiotocographic monitoring into protocols for management of high-risk pregnancy in the United States. Fetal arousal tests such as vibroacoustic stimulation have largely been evaluated in high-income countries. There is a need for further studies in LMICs with limited CTG facilities [79].

##### Assessment

There are surprisingly few studies evaluating the relationship of cardiotocographic monitoring with stillbirth and the overall quality of available evidence is low. In light of the probable link with improved outcomes in developed countries, and the widespread use of low-cost CTG monitoring equipment in clinical practice, the current recommendation is weak. Further studies must be conducted with appropriate design and power to assess this intervention in health systems in developing countries.

#### Doppler and late ultrasound monitoring

Doppler ultrasound is a technique to study the fetoplacental and/or uteroplacental circulatory dynamics. A Cochrane review included 11 RCTs comparing umbilical artery Doppler ultrasound in complicated pregnancies with no Doppler, and found a nonsignificant 21% reduction in the stillbirth rate (OR 0.79, 95% CI 0.461.34) [80]. A similar review of late ultrasound examination for all pregnancies - whether or not these were complicated - showed no effect [81].

##### Assessment

There is high-quality evidence suggesting a lack of effect on stillbirth, and the recommendation against this intervention is strong.

### Interventions for pregnancy infections

#### Screening and treatment of syphilis

Syphilis during pregnancy is common in many LMICs, with prevalence in local studies varying widely from less than 1% to 10% or higher [82]. African studies show prevalence during pregnancy of 3.4% in Uganda, [83] 7.7% in Tanzania, [84] 12% in Malawi, and 17.4% in Cameroon [85]; [86]. Syphilis produces villitis and obliterative arteritis, which are severe lesions in the placenta associated with fetal and newborn mortality [87].

##### Preterm birth

During pregnancy, observational studies show associations between syphilis and both preterm birth and low birth weight. In Tanzania, women with high-titer active syphilis had a six-fold greater risk of preterm birth and a three-fold increase in low birth weight compared with seronegative women. It was estimated that syphilis accounts for one fourth of the preterm births in this population [88]. An earlier study from Malawi also found an increase of preterm and low birth weight babies among women with syphilis (OR 3.6; 95% CI 1.6-7.9) [89]. All studies reviewed are from LMICs.

Penicillin effectively reduces the risk of congenital syphilis [84,90], but there are no intervention studies showing an effect of syphilis screening and treatment on preterm birth.

##### Stillbirth

The effectiveness of antibiotics in curing gestational syphilis and preventing congenital infection was established in the 1940s, before RCTs had been adopted [90]. Observational studies in Swaziland, and Kenya suggest syphilis screening and treatment is associated with reduced perinatal mortality [91] and reduced stillbirths [84,92]. In the Tanzanian study [84], the proportion of stillbirths was higher among women treated for low-titer active syphilis (4.8%) vs. seronegative women (2.5%) (crude OR = 1.95, 95% CI: 0.96 - 4.0); being similar for women treated for high-titer active syphilis (2.3%) versus seronegative ones (2.5%). For ethical reasons it was not possible to have an untreated group with syphilis, but these results suggest that treatment of high-titer women can reduce the risk. The results for low-titer women are difficult to interpret in the absence of an untreated comparison group.

##### Assessment

The quality of evidence for preventing preterm birth, low birth weight and stillbirth is moderate, being based on observational studies and accumulated knowledge. Given the current level of knowledge, it is not expected that RCTs will be conducted as these would be unethical. The recommendation is weak in favor of the intervention for preventing preterm birth or low birth weight. The recommendation is strong in favor of recognizing and treating maternal syphilis to reduce stillbirth and congenital syphilis.

#### Intermittent presumptive treatment during pregnancy (IPTp) (for malaria)

Malaria is a key cause of maternal illness and anemia in pregnancy, especially among primiparae in areas where malaria is endemic such as sub-Saharan Africa and parts of Latin America and Asia. Malaria prevention includes the use of antimalarial drugs administered presumptively through strategies such as intermittent preventive treatment during pregnancy (IPTp) and the use of insecticide-treated bed nets (ITNs).

##### Preterm birth

Malaria infection may affect fetal growth and gestational duration through maternal anemia and placental infection [93]. A systematic review of interventions with antimalarials during pregnancy showed that - among women in their first or second pregnancies - treatment reduced anemia, parasitaemia, placental malaria, perinatal deaths and low birth weight (six trials, RR 0.57; 95% CI 0.46-0.72). No effect on preterm births was observed in the only trial assessing this outcome [94].

##### Stillbirth

A Cochrane review on prophylactic antimalarials or IPTp showed no overall effect, but when the analyses were restricted to women in their first or second pregnancy, antimalarials significantly reduced perinatal mortality (RR 0.73; 95% CI 0.53-0.99) and were associated with a smaller, nonsignificant reduction in stillbirths (RR 0.87; 95% CI 0.62-1.21) [94].

##### Assessment

Although there is little evidence for preterm birth or stillbirth, the quality of evidence is high for using IPTp to reduce low birth weight and perinatal mortality in malaria- endemic areas. IPTp is thus strongly recommended for women in their first or second pregnancy.

#### Insecticide-treated mosquito nets (ITNs)

ITNs are known to decrease malaria transmission and reduce mortality [95]. Nevertheless, it is estimated that only a small fraction of women and children living in malarious areas are currently protected [96].

##### Preterm birth

A review of five randomized clinical trials of ITNs during pregnancy—four in Africa and one in Thailand—showed a 33% reduction in low birth weight in the African trials, but no effect in Thailand [97]. In the only trial with information on preterm births, conducted in Kenya [98], no effect was demonstrated.

##### Stillbirth

The same Cochrane review showed a 33% reduction in fetal losses (abortions and stillbirths) in the first to fourth pregnancy (RR 0.67; 95% CI 0.47-0.97), but not in fifth and higher-order pregnancies [97,99].

##### Assessment

The quality of evidence is high for low birth weight and stillbirth, and the intervention is strongly recommended for these outcomes. There is very limited evidence—a single RCT—for preventing preterm birth, and the recommendation is weak (against), given that this trial found no effect. Overall, ITNs are strongly recommended due to their effect on maternal and child mortality and morbidity. There are few studies employing a combination of IPTp and ITN on pregnancy outcomes, including preterm birth and stillbirth.

#### Screening and treatment of asymptomatic bacteriuria

Genital and urinary infections, including asymptomatic bacteriuria, may affect preterm birth. One possible pathway could be a direct intrauterine infection, but it is also possible that the infection produces an inflammatory reaction that leads to preterm birth, even after the infection has been treated [39].

##### Preterm birth

Observational studies show an association between maternal urinary tract infections and both preterm birth and low birth weight. A meta-analysis of four observational studies indicated a decreased risk of preterm birth (RR 0.51, 95% CI 0.36-0.69) in nonbacteriuric pregnant women in comparison to those with bacteriuria [100].

A 2007 meta-analysis evaluated 14 RCTs comparing the effect of antibiotics versus placebo [101] on different outcomes. Treatment reduced maternal pyelonephritis (RR 0.23; 95% CI 0.13-0.41) and low birth weight (7 studies; RR 0.66; 95% CI 0.49-0.89), but did not significantly reduce preterm birth (3 studies; OR 0.37; 95% CI 0.10-1.36). The three studies addressing preterm birth, however, had a number of methodological problems, such as using different cutoff points (<38 weeks in two studies and <37 weeks in one), having small sample sizes, and different diagnostic and treatment criteria.

##### Stillbirth

No reported outcomes.

##### Assessment

The quality of evidence for preterm birth is low, but it is high for low birth weight. The intervention is strongly recommended for preventing low birth weight and reducing maternal morbidity, but has a weak recommendation for preventing preterm births. Further studies are required on preterm birth and stillbirth.

#### Screening and treatment of bacterial vaginosis

As is the case for bacteriuria, bacterial vaginosis might contribute to preterm labor through infectious or inflammatory mechanisms [39]. Pregnant women with bacterial vaginosis are two to three times more likely to have a preterm birth than women without vaginosis [39]. It is not clear, however, whether this is a causal association [102].

##### Preterm birth

Several systematic reviews of screening and treatment for bacterial vaginosis found no impact on preterm births or low birth weight [103-108]. Because of this, routine pregnancy screening and treatment of bacterial vaginosis is not recommended [109-111].

However, RCTs in which antibiotics were given before 20 weeks found a significant reduction in preterm births (RR 0.72; 95% CI 0.55-0.95) [107]—but further trials are needed before a recommendation for early treatment can be issued. It is possible the bacteria that cause the infection may ascend into the uterus before or during early pregnancy, starting an inflammatory response that would not be affected by late treatment [39].

##### Stillbirth

No reported outcomes.

##### Assessment

There is high quality evidence the treatment of bacterial vaginosis has a lack of effect for preventing preterm birth or low birth weight. A strong recommendation is made against the use of the intervention. The effect of early treatment of bacterial vaginosis on preterm birth and stillbirth is a research gap that needs to be addressed.

#### Prevention of mother-to-child transmission of HIV

In high-income countries, highly active antiretroviral therapy (HAART) has markedly reduced vertical transmission rates of HIV. However, there is concern that preterm rates may have increased among treated women. Analyses have yielded inconsistent results with respect to pregnancy outcomes. In particular, it is uncertain whether or not combination ART—most specifically that which includes protease inhibitors (PI's)—is associated with an increase in preterm births.

##### Preterm birth

Observational studies, particularly those from Europe, report increased rates of preterm birth in women receiving HAART during pregnancy. This risk was particularly pronounced for PI use that started early in pregnancy or before conception. For example, a multicenter European collaborative study including nearly four thousand mothers reported relative risks of preterm birth of 2.60 (95% CI 1.43-4.75) and 1.82 (95% CI 1.13-2.92), for those exposed to combination therapy with and without a protease inhibitors, respectively, compared to no treatment [112]. An observational study in the USA also showed increased rates of preterm birth among women receiving HAART with protease inhibitors, compared with any other treatment option (OR 1.8; 95% CI 1.1-3.0) [113]. However, a 2007 meta-analysis of 1 retrospective and 13 prospective studies, including the aforementioned, showed that antiretroviral therapy during pregnancy is not associated with an overall increased risk of preterm birth [114]. The use of combination regimens before or early in pregnancy may slightly increase the risk of preterm birth, although confounders such as maternal HIV-stage may contribute to this observation. Observational studies suggest an association between preterm birth and use of HAART in pregnancy, in particular initiating PI-based HAART early in gestation, but inadequately control for stage of maternal HIV disease and other potential confounding variables [115-118].

##### Stillbirth

Fewer studies report on stillbirth outcomes and antiretroviral therapy during pregnancy. A report combining two completed clinical trials and five ongoing, prospective observational studies from the United States found similar rates of stillbirths between women who did and did not receive antiretroviral therapy during pregnancy [119]. Follow-up analysis from 2 of the included observational studies continued to show either no association between ART and stillbirth [113] or a decreased risk for stillbirth (OR 0.06, 95% CI 0.02-0.18) [120]. Townsend et al., 2007 compared women on HAART with women on mono/dual therapy. In comparison with exposure to mono/dual therapy, exposure to HAART was associated with a nonsignificant increased risk of stillbirth (adj. OR=2.27, 95% CI: 0.965.41; p=0.063) [121].

Finally, there was no significant difference in stillbirth rates between groups of women treated with HAART throughout pregnancy and short-course therapy in the two studies originating from low-income countries [122,123].

##### Assessment

The use of combination antiretroviral therapy has dramatically decreased the rate of maternal-to-child HIV transmission. However, observational data suggest a possible association between ART therapy and increased risk of preterm birth. This may be particularly burdensome in low-income countries, particularly those with less access to neonatal care. Large-scale, randomized, controlled trials are needed to define the risk of HAART therapy and adverse pregnancy outcomes.

#### Anti-helminthic treatment

Hookworm infestation is associated with anemia in women and children in endemic areas. In such areas, routine antenatal mebendazole therapy could greatly reduce the prevalence of anemia in pregnancy.

##### Preterm birth

One RCT in Peru found no difference in low birth weight between women receiving anti-helminthitic therapy (mebendazole) compared to placebo (8.1% and 8.7%, respectively; p=0.7). Both groups received iron supplements. No information on gestational age was available [124]. A recent Cochrane review of the treatment of soil-related helminths in pregnancy shows no statistically significant impact on preterm birth (RR 0.85, 95% CI 0.38-1.87) or low birth weight (RR 0.94, 95% CI 0.61-1.42) [125].

##### Stillbirth

An observational study from Sri Lanka on the effect of mebendazole therapy during pregnancy on birth outcomes showed that stillbirths and perinatal deaths combined were significantly less common among women who received mebendazole as part of antenatal care (RR 0.55; 95% CI 0.4-0.77) [126]. However, the Peruvian RCT did not show statistically significant differences in rates of miscarriage, stillbirth (8/522 in the mebendazole plus iron group vs. 4/520 in placebo plus iron) or early neonatal death [127], but was underpowered for these outcomes.

##### Assessment

The quality of evidence is low, and there is a weak recommendation against using anti-helminthics to prevent stillbirth. Very little information is available on preterm birth. Hookworm treatment, however, is recommended for pregnant women to reduce anemia in high-prevalence populations.

#### Screening and treatment for periodontal disease

Periodontal disease has been associated with preterm birth, but the biological pathway for this association is not known. One possible explanation may be gingival infection resulting in intrauterine infection, via maternal bacteraemia and transplacental passage. This relationship is not proven [39].

##### Preterm birth

A meta-analysis of observational studies indicate associations between periodontal disease and preterm birth [128], but clinical trials show conflicting results [129]. From the four available RCTs, two showed no effect of treatment on preterm birth or low birth weight [130]. The other two described reductions in preterm birth, and one RCT showed a reduction in low birth weight [131,132].

##### Stillbirth

No reported outcomes.

##### Assessment

Although RCTs on the impact of periodontal treatment on preterm birth or low birth weight are available, the quality of the evidence is moderate, because results so far are inconclusive. Currently, the intervention is not recommended for the sole intent of preventing preterm birth or low birth weight. The intervention may be beneficial for improving maternal oral health, but further research on fetal outcomes is needed.

### Interventions for pregnancies with high-risks of preterm birth or stillbirth

#### Progesterone

Progesterone has been proposed for preventing the recurrence of preterm birth. The possible mechanisms supporting its use include: anti-inflammatory action, oxytocin antagonism (producing relaxation of smooth muscle), maintenance of cervical integrity, and reduced gap-junction formation [109].

##### Preterm birth

A meta-analysis of six randomized trials comparing the use of progesterone with placebo in high-risk women, showed a reduction of preterm births in the intervention group (RR 0.65, 95% CI 0.54-0.79) [133], as well as a decreased prevalence of low birth weight (four studies, RR 0.63, 95% CI 0.49-0.81). Five of the six trials are from high-income settings.

##### Stillbirth

The same meta-analysis failed to show a significant impact on perinatal deaths (five studies, RR 0.66; 95% CI 0.37-1.19).

##### Assessment

The evidence of progesterone use to prevent recurrence of preterm birth is high and the intervention is strongly recommended. For stillbirth, the evidence is moderate due to the wide confidence interval, and the recommendation is weak against this intervention.

#### Cervical cerclage

Cervical cerclage is used for the treatment of an incompetent cervix that is associated with previous spontaneous abortion or miscarriage.

##### Preterm birth

In four RCTs of cervical cerclage for high-risk women, reviewed in a Cochrane publication [134], there was no difference in the occurrence of preterm births between intervention and control women (RR 1.04, 95% CI 0.99-1.10).

##### Stillbirth

Another Cochrane review showed a nonsignificant 20% reduction in perinatal loss, death at or after 24 weeks of gestation and up to the first week of neonatal life. (RR 0.80, 95% CI 0.48-1.36) [135]. A more recent systematic review of seven RCTs also found similar results for pregnancy loss or death before hospital discharge (OR 0.81, 95% CI 0.60-1.10) [136].

##### Assessment

The quality of evidence is high for both preterm birth and pregnancy loss. The recommendation is strong against the intervention for preventing preterm delivery, and weak against for preventing stillbirth, due to greater uncertainty in the evidence.

#### Multivitamins for HIV+ women

Poor maternal micronutrient status has been associated with faster clinical and immunological evolution of HIV disease. This association led to research on vitamin supplementation [137].

##### Preterm birth

A systematic review of four RCTs comparing the use of vitamin A with placebo in HIV-positive pregnant women did not show significant effects on maternal-to-child HIV transmission (OR 1.06; 95% CI 0.89-1.26) or preterm birth (RR 0.88, 95% CI 0.65-1.19). The intervention significantly increased mean birth weight by 89 g (95% CI 85-95) and resulted in a near-significant reduction in low birth weight (RR 0.83, 95% CI 0.68-1.01) [138].

##### Stillbirth

Only one study reported fetal loss (miscarriage and stillbirth) and indicated a reduction in stillbirth with multi-vitamin supplementation among HIV-infected women in pregnancy (30/512 in supplemented groups versus 49/509 in controls (RR 0.61, 95% CI 0.39-0.94) [137].

##### Assessment

The quality of evidence for preterm birth and low birth weight is moderate due to uncertainty in the estimates, whereas the quality is low for stillbirth given that only one study is available. The recommendation is weak against this intervention.

### Intrapartum interventions to prevent stillbirth

Intrapartum deaths account for a third of all stillbirths globally. Interventions to reduce such deaths include acting on the demand side by helping families seek care from skilled attendants, and acting on the supply side by improving access to and the quality of skilled care. These interventions are reviewed in terms of their potential impact on stillbirth and other perinatal outcomes, as intrapartum procedures are not carried out with the objective of affecting preterm birth.

#### Birth preparedness

Birth preparedness consists of preparing the mother, family and community for delivery and potential complications. It includes several measures such as seeking appropriate care during pregnancy, identifying the place of delivery, acquisition of sterile materials and planning for skilled birth attendance and referral if needed—including setting aside money and arranging for transportation to a facility [139].

In recent years many community based trials in Asia have employed various cadres of community health workers and support groups to promote birth preparedness and effective newborn care [140-143]. Such community-based interventions can play an important role in promoting birth preparedness, especially in seeking emergency obstetric care. However, existing studies include birth preparedness as part of a package of several other antenatal and delivery interventions, so it is not possible to separate the impact of birth preparedness alone.

##### Assessment

There is high quality evidence that community-based interventions that include birth preparedness can prevent stillbirths. However, the effects of birth preparedness per se cannot be assessed. Community-based interventions are strongly recommended in appropriate settings where the proportion of home deliveries is high.

#### Use of partogram

A partogram, also called a partograph, is a simple preprinted paper form on which midwives and obstetricians can record the progress of labor. The tool provides a continuous pictorial overview of the progress of labor, and distinguishes between the latent and active phases of labor. Slower progress identified by the alert line on the partograph can be used as a basis for transfer to a facility for skilled intervention and delivery.

The recent Cochrane review by Lavender et al. 2008 [144] showed no statistically significant effect of partogram use on the Caesarean section rate (RR 0.64, 95% CI: 0.24-1.70), instrumental vaginal delivery (RR 1.00, 95% CI: 0.85-1.17) or Apgar score <7 at 5 minutes (RR 0.77, 95% CI: 0.29 - 2.06). There was no perinatal mortality in the two groups (partograph with 2-hour action line vs. 4-hour action line) in studies in high-resource settings [145,146] included in this review. A large, multicenter study from Southeast Asia [147] reported a stillbirth rate of 0.3% among women using the partogram vs. 0.5% for the control group.

##### Assessment

The quality of evidence reviewed for evaluating the partogram for care during delivery is low, given the controlled circumstances in which these studies have been conducted. The recommendation is weak in favor of this intervention. Further RCTs are required in which stillbirth is a primary outcome.

#### Fetal movement monitoring

Reduced fetal movements are associated with a higher risk of stillbirth, [148] and fetal movement records or kick charts have been proposed as a screening mechanism. However, a large RCT comparing the impact of the use of kick charts on unexplained stillbirths found no difference in the rate of fetal death between the intervention (2.9/1000) and control group (2.7/1000) [149]. Another study comparing fetal monitoring with hormonal profiling in 1191 pregnant women [150] was inconclusive (RR 3.19, 95% CI 0.13-78.2).

##### Assessment

Only two studies are available on this intervention, both from developed countries, with no significant effects on stillbirth. As a consequence, the quality of the evidence is low and there is a strong recommendation against its adoption.

#### Emergency obstetric care

Many intrapartum stillbirths can be prevented with improved obstetric care, including emergency cesarean sections (c-sections). It is estimated that no fewer than 5% of all deliveries require a c-section due to maternal or fetal indicators [151,152]. These operations gained wide acceptance several decades ago, and therefore randomized trials are not available regarding their overall effect on stillbirths. Ecological analyses using countries as the data units show that, in low and middle-income countries, each one percentage point increase in the c-section rate is associated with a reduction of 1.6/1,000 in the intrapartum stillbirth rate; this is observed for c-section rates between 0 and 8%, after which the relationship flattened out [153].

Instrumental deliveries using forceps or vacuum extraction account for 5 - 20% of all births in most high- income countries [154]. As is the case for c-sections, there are no RCTs of instrumental versus non-instrumental approaches for complicated deliveries, but trials comparing different types of instruments are available. A Cochrane review of seven RCTs comparing vacuum versus forceps reported a nonsignificant difference in perinatal mortality rate in the two methods (OR 0.80; 95% CI 0.18-3.52), with extremely wide confidence limits [155]. Other studies also reported similar neonatal outcomes in the two groups [156]; [157].

##### Assessment

The quality of evidence in favor of c-sections or instrumental deliveries, compared to no such procedures, is moderate for stillbirths. Fifteen of the 40 studies reviewed on this topic were carried out in LMICs. RCTs for testing these approaches would be unethical. The recommendation is strong in favor of the interventions to prevent stillbirths. Excessively high c-section rates, however, should be avoided, as the frequency of preterm delivery and neonatal mortality both rise at rates of caesarean delivery of between 10% and 20% [158].

#### Cesarean section for breech presentation

Around 3-4% of term singleton pregnancies are complicated by breech presentation. A total of six studies were identified, three of which were from LMICs. A Cochrane review of three RCTs comparing planned cesarean with planned vaginal delivery for breech infants showed a 71% reduction in perinatal or neonatal death, excluding fatal malformations (RR 0.29; 95% CI 0.10-0.86) [159].

##### Assessment

The quality of evidence for preventing perinatal mortality is high and the recommendation for this intervention is strong.

#### Elective induction of labor for post-term delivery

Induction of labor is advocated when vaginal delivery is the appropriate route of delivery and gestational age is 41 completed weeks or more. The Cochrane review comparing induction with expectant management consisting of twelve trials in low-risk women with intact membranes showed a nonsignificant reduction in stillbirths (RR 0.28; 95% CI 0.05-1.67) and a significant impact on perinatal mortality (RR 0.30; 95% CI 0.09-0.99) [160]. Only one of the 15 studies identified, which included controlled trials and observational studies, was from an LMIC.

##### Assessment

The quality of evidence for elective induction for post-term pregnancies is high for perinatal mortality, and the recommendation is strong in favor of the intervention in low-risk pregnant women at 41 weeks or more.

#### Elective induction for women with term premature rupture of membranes (PROM)

Another Cochrane review summarized the effect of induction in women with term, premature rupture of membranes. The pooled results of five RCTs suggest a reduction in fetal or perinatal mortality (OR 0.46; 95% CI 0.13-1.66) [161]. No stillbirth outcomes were reported separately. Although this difference is not significant, there were significant improvements in other maternal and infant morbidity indicators.

##### Assessment

The quality of evidence for perinatal mortality is moderate given the wide uncertainty of the estimates, and the recommendation is weak in favor of induction. However, the recommendation is strong for improving other maternal and infant outcomes.

#### Home delivery versus facility births

Mothers in low- and, to a lesser extent, middle-income countries often do not have a choice about where to deliver their babies due to limited access to hospital care. The evidence from RCTs of home versus institutional birth is therefore derived from high-income countries. A Cochrane review [162] found a near-significant increase in risk of perinatal mortality (five trials; RR 1.83; 95% CI 0.99-3.38) in home-like settings, compared to hospital deliveries. In Australia, an observational study [163] reported a significant reduction in perinatal mortality in delivering at 'alongside hospital' birth centers compared to hospital deliveries, but these results may have been affected by selection bias.

##### Assessment

The quality of evidence for perinatal mortality is moderate, and the recommendation is weak against home delivery.

### Intrapartum interventions to improve preterm survival

This section reviews interventions for the mother when preterm labor has been initiated but the newborn has not yet been delivered. The main objective of these interventions is to improve the survival of preterm newborns.

#### Prophylactic corticosteroid therapy in preterm labor

Several agencies recommend that women in preterm labor before 34 weeks and those with preterm rupture of membranes (PROM) under 32 weeks should receive a single dose of either betamethasone or dexamethasone [164-166]. A Cochrane review showed use of corticosteroids in preterm labor reduced respiratory distress syndrome by 36% (RR 0.64, 95% CI 0.56-0.72), with a maximum effect at 32 weeks of gestation. Steroids also reduced cerebral hemorrhage by 70% (RR 0.30; 95% CI 0.14-0.66), and neonatal mortality by 37% (RR 0.63; 95% CI 0.51-0.77) [167]. Four of the 21 studies reviewed are from LMICs.

##### Assessment

The quality of evidence for preventing preterm birth, morbidity and mortality is high and the intervention is strongly recommended.

#### Antibiotics for preterm labor with premature rupture of membranes (PROM)

Premature rupture of membranes is strongly associated with infection of the amniotic membranes, and this infection is independently related to preterm birth, [168] cerebral palsy and chronic lung disease [169]. A Cochrane review showed antibiotic treatment for PROM led to reductions in the proportion of babies born within 48 hours (RR 0.71; 95% CI 0.58-0.87), and reduced neonatal infections (RR 0.68; 95% CI 0.53-0.87), surfactant use (RR 0.83; 95% CI 0.72-0.96), oxygen therapy (RR 0.88; 95% CI 0.81-0.96), and abnormal cerebral ultrasound scans prior to hospital discharge (RR 0.82; 95% CI 0.68-0.98) [170]. No differences in long-term follow-up were observed in the babies of intervention and control groups [171]. Five of the 22 studies reviewed are from LMICs.

##### Assessment

The quality of evidence on morbidity and mortality is high and the intervention is strongly recommended to be scaled up to improve preterm survival.

#### Antibiotics for preterm labor with intact membranes

Meta-analyses of RCTs of the use of antibiotics for preterm labor with intact membranes did not show improvements in preterm birth or in preterm morbidity (RR 1.03; 95% CI 0.86-1.24) [172,173]. In addition, in a seven year follow-up study, increased functional impairment was described in children whose mothers received erythromycin during labor [174].

##### Assessment

The quality of evidence is high, and there is a strong recommendation against antibiotics for preterm labor with intact membranes.

#### Early versus delayed cord clamping in preterm newborns

Immediate umbilical cord clamping, often within 15 seconds after delivery, is the current practice in most settings. A limited number of observational trials, most of which were conducted prior to 1980 [175-180] have given way to this belief. These trials reported higher rates of complications in preterm newborns with delayed cord clamping [181].

Two recent meta-analyses of outcomes among preterm newborns [182,183] have challenged the benefit of immediate cord clamping. Benefits of delayed cord clamping included higher circulating blood volume during the first 24 hours of life, less need for blood transfusions and lower incidence of intraventricular hemorrhage (RR= 0.53, 95% CI= 0.35 - 0.79). There was also no significant increase in the risk of respiratory distress syndrome (RR= 0.83, 95% CI= 0.59 - 1.15) or necrotizing enterocolitis (RR= 2.08, 95% CI= 0.52 - 8.37). Early cord clamping was associated with a nonsignificant increased mortality risk in babies compared to delayed clamping (RR1.40, 95% CI 0.59-3.32, p=0.45).

Four recent additional RCTs [184-187] were identified out of which only one study [186] reported relevant outcomes, showing no differences in mortality rates (p=1.00), intraventricular hemorrhage (p=0.67) or necrotizing enterocolitis (p=1.00) between the early and delayed cord clamping groups.

##### Assessment

On the basis of available evidence, a 30-second delay in cord clamping is a safe intervention for preterm newborns. The quality of the evidence is high and the recommendation is strong in favor of this intervention. All studies are from high-income countries.

### Postnatal interventions to improve preterm survival

This section reviews interventions aimed at improving the management of preterm newborns to reduce neonatal mortality.

#### Neonatal resuscitation

About 5-10% of newborns require some form of resuscitation ranging from simple maneuvers to assisted ventilatory support [188]. Resuscitation is a difficult intervention to review given the obvious constraints in study design and locations. Given the paucity of data and limited studies in preterm newborns, we have also included relevant summary information from studies undertaken among term newborns and have summarized the information from preterm newborns, where available. The final recommendations are thus based on the biological plausibility and feasibility of the intervention.

The literature reviews four main areas of research regarding resuscitation:

##### 1. Modes of oxygen delivery and resuscitation techniques

Different approaches have been used for oxygen delivery to the neonate (face mask, bag mask, nasal cannulae, CPAP) and these may vary in terms of achieving adequate oxygen saturation and hence dictate the need for further management (intubations, CPR etc). Only one study addressed preterm neonates exclusively [189] while others reported outcomes in all newborns.

Capasso et al [190] compared oxygen delivery on intermittent positive pressure with nasal cannulae versus facial mask in primary resuscitation of the newborn with moderate asphyxia, concluding that while resuscitation with nasal cannulae required fewer intubations and chest compressions, it had comparable survival rates and Apgar scores to normal controls. Palme et al [191] compared the efficiency of five widely used face masks on newborns with respect to the rates of leakage and frequency and ease of cleaning. They reported least leakage with circular silicone rubber mask ('Laerdal') and found it easier to clean, boil and autoclave.

Massawe et al [192] found that mouth-to-mask and bag-to-mask ventilation were comparable in terms of Apgar scores, heart rate and time to first breath. The only RCT on preterm newborns [189] evaluated the impact of nasal CPAP on BPD and the need for intubation, reporting a lower number of intubations in neonates given nasal CPAP on admission to the NICU compared to those who were not given nCPAP.

These results suggest that nasal CPAP is preferable to manual inflations in preterm newborns and may lower the need for chest compressions and intubations, whereas mouth-to-mask ventilation is equivalent to bag-to-mask ventilation for neonates. The American Academy of Pediatrics has initiated a Helping Baby's Breathe program (AAP, personal communication 2009) to enable lower level health workers to undertake standardized resuscitation in primary care settings. Validation studies of the protocol are underway.

###### Assessment

As for CPR, while guidelines for resuscitating preterm newborns exist there are a number of areas of uncertainty which require further research and refinement. On the basis of moderate-quality evidence, it appears that bag and mask resuscitation is adequate in most circumstances.

However, further research is needed to establish the adequacy of this form of resuscitation in community settings in well designed, large scale studies.

##### 2. Use of cardio-pulmonary resuscitation (CPR) techniques and its long-and short-term outcomes

The crucial role of neonatal resuscitation in immediate newborn care is well accepted [193]. On the other hand, its use has also been associated with adverse long- and short-term outcomes possibly due to survival of severely affected newborns [194-196]. Given that RCTs of such interventions are difficult, we reviewed seven observational studies from PubMed and one interventional study from Cochrane clinical trials. Segregated data for term and preterm neonates was available for two studies, whereas other authors did not separate these two groups.

Vakrilova et al [197] discussed the need of delivery room cardio-pulmonary resuscitation (CPR) in VLBW and ELBW neonates and reported that birth weight and gestational age are the most important factors, determining the intensity of delivery room CPR and the prognosis in newborns of < 1500 g. Multiple studies reported the long-term outcomes including language symmetry, attention shift, visual attention, neuropsychological sequela and social and educational adjustments. While language symmetry and attention shift [196] were impaired in the individuals given CPR at birth, there was no difference between the resuscitated and non resuscitated groups in terms of other parameters for long-term outcomes [195,196,198,199]. Dorfsman et al [200] compared two thumb technique of neonatal CPR versus American Heart Association recommended two finger technique. He reported that TT (two thumb) chest compression produced higher MAP, systolic and diastolic blood pressures when compared with TF (two finger) chest compression technique during a clinically relevant duration of prolonged CPR.

Sanchez-Torres et al [194] evaluated the impact of CPR provided in delivery room on survival and short term neurological outcomes in preterm infants. The infants receiving CPR and those infants who did not were comparable in terms of mortality, NEC, IVH and BPD. However the infants undergoing CPR needed surfactant and oxygen more frequently. On the other hand Deulofeut et al [201] reported higher rates of mortality, IVH and periventricular leucomalacia in neonates who were provided CPR at birth.

###### Assessment

While it is entirely appropriate to resuscitate preterm newborns, there are a number of issues that require further research as the current quality of the evidence is low. These include appropriate techniques and duration for CPR in preterm newborns and understanding intermediate- and long-term outcomes.

##### 3. Room air (versus 100% oxygen) for resuscitation

The optimum oxygen concentration required for newborn resuscitation has been the subject of investigation [202]. While 100% oxygen has been traditionally used for all cyanotic infants [203], reports of increasing incidence of hypoxic ischemic encephalopathy (HIE), as well as other secondary outcomes, have made its use debatable. High oxygen levels were also associated with adverse outcomes like high oxidative stress [204] and increased incidence of leukemia [205].

It has been proposed that room air is as effective as 100% oxygen in overcoming neonatal asphyxia, [188] and several studies and systematic reviews addressed this issue [202,203,206]; [188,204,207-210].

Three intervention studies on preterm infants [211-213], all from high-income settings, reported similar mortality and morbidity rates, including BPD, among those resuscitated with room air or 100% oxygen (p=0.95 for NMR) [211], p=0.71 for NMR [212].

Studies which did not separate term and preterm newborns compared 100% oxygen with room air, concluding either that outcomes are similar in both groups, or that the room air group has advantages in terms of morbidity and mortality [202,203,206]. Saugstad et al [214] also reported no differences in neurodevelopmental outcomes between neonates resuscitated with room or 100% oxygen and this finding has been replicated in a recent population based study by Hellstrom-Westas *et al.*[215].

###### Assessment

These studies indicate the use of room air is comparable to 100% oxygen with respect to primary neonatal outcomes with some ostensible benefits. The overall quality of evidence is high and this intervention can be recommended as a standard approach to resuscitating preterm neonates.

##### 4. Provision of facilities and training health professionals in neonatal resuscitation

Many studies confirm that training programs for neonatal resuscitation are vital for survival [193,216,217].

Impact on preterm infants was observed in three studies where training programs led to significant reductions in terms of mortality and morbidity [218-220]. Specialized neonatal resuscitation teams have also played an important role in reducing preterm neonatal morbidity and mortality [221].

Additional studies, mostly observational, document the impact of training programs on all newborns, either term or preterm. There is ample documentation on the impact of such programs on improving the performance and technical abilities of health professionals, and on reducing neonatal morbidity and mortality rates [219,222-239]. Similar outcomes were also observed by the employment of specialized neonatal resuscitation teams [221,240,241].

Finally, large-scale studies support the effectiveness of resuscitation programs. Sen et al [217] demonstrated a 21% reduction in mortality after a sick newborn care unit was set up in Kolkata that included resuscitation facilities. Enweronu-Laryea et al [242] reported similar effect of improved neonatal care facilities on neonatal survival in Ghana which provided improved obstetric services, referral systems, well developed neonatal units and trained staff.

###### Assessment

While specific data on the benefit of resuscitation training programs on survival of preterm infants is limited, it is plausible that adequately trained health professionals and neonatal resuscitation programes are also effective in reducing asphyxia-related preterm mortality as well as reducing complications such as neurodevelopmental delay, NEC and BPD. Given the large number of observational studies carried out under diverse settings, the quality of the evidence is judged to be moderate. This intervention is recommended with the provision that these outcomes be evaluated in large-scale effectiveness settings.

#### Vitamin A supplementation in preterm neonates

Vitamin A is involved in the growth and differentiation of various body cells including those of the respiratory, gastrointestinal and immune systems. Preterm infants are deficient in vitamin A due to inadequate hepatic stores and the inability to tolerate routine oral supplementation [243,244].

A 2007 Cochrane review of very low birth weight infants showed a significant but small reduction in death or oxygen use at one month [RR= 0.93; 95% CI 0.88, 0.99] in infants receiving intravenous vitamin A compared to placebo [243]. There were also nonsignificant reductions in nosocomial infection and neurodevelopmental delays at 18 to 36 months of age.

The recent Lancet Nutrition series identified neonatal vitamin A supplementation in South Asia were associated with a significant reduction in infant mortality at 6 months [245]. From these recent trials of neonatal vitamin A supplementation, it was possible to obtain separate data for preterm infants from three RCTs [246-248]. and to conduct an additional meta-analysis. Neonatal vitamin A supplementation among preterm infants was associated with a nonsignificant reduction in infant mortality at 6 months of age (RR 0.87; 95% CI 0.65, 1.17).

##### Assessment

The quality of evidence is moderate given the small number of studies including preterm infants and the uncertainty in estimates. WHO has recently commissioned three large prospective RCTs in community settings to evaluate the impact of neonatal Vitamin A supplementation. Given current knowledge, there is a weak recommendation against its use in preterm infants, but this may well change as the evidence accumulates.

#### Antenatal and postnatal vitamin K supplementation in preterm infants

Vitamin K is a fat-soluble vitamin crucial to the production of many proteins involved with the coagulation process. Since 1961 the Committee on Nutrition of the American Academy of Pediatrics has recommended that prophylactic vitamin K be administered parenterally to all newborns, primarily for preventing intracranial hemorrhage [249].

Vitamin K treatment of pregnant mothers before preterm delivery has also been proposed. A Cochrane review [250] evaluated five randomized or quasi-randomized trials of vitamin K administered parenterally or orally to women at risk of imminent preterm birth, none of which were from LMICs. The primary outcomes were neonatal mortality, neonatal neurological morbidity, as measured by the presence of periventricular haemorrhage (PVH) on ultrasound during the first week of life, and long-term neurodevelopment. Antenatal vitamin K was associated with a nonsignificant reduction in all grades of PVH (RR 0.82, 95% confidence interval (CI) 0.67-1.00) and in severe PVH (grades 3 and 4) (RR 0.75, 95% CI 0.45-1.25) for babies receiving prenatal vitamin K compared with control babies. In a subsequent RCT by Liu et al [251], 90 pregnant women in preterm labor at less than 35 weeks of gestation received vitamin K1 10 mg per day injection intramuscularly or intravenously for 2-7 days, or no such treatment. The overall rates of PVH were 32 and 52%, respectively (p=0.036), and the frequency of severe PVH was 5 and 20%, respectively (p=0.038). Although the quality of the trials is variable and further studies are needed, these data suggest a possible role for antenatal maternal vitamin K administration as a means of reducing risks of PVH and mortality in preterm infants.

Evidence supporting the use of injectable vitamin K (phytomenadione) shortly after birth to prevent hemorrhage-related morbidity and mortality was accumulated before RCTs were widely adopted, being based on observational studies. The strongest evidence from high-income countries comes from periods in which concerns about the safety of vitamin K led to its reduced use in newborns; these periods were followed by increased incidence of hemorrhagic disorders [252].

However, the exact dose requirement and frequency in preterm infants remain uncertain. Due to ethical reasons recent RCTs did not include a placebo group, but compared different treatment modes, using vitamin K status as the primary outcome. [253,254].

##### Assessment

The quality of the evidence for both antenatal and postnatal vitamin K use is moderate. There is a strong recommendation for postnatal vitamin K, and a weak recommendation in favor of antenatal vitamin K for women in premature labor.

#### Postnatal zinc supplementation in preterm infants

Zinc plays an important role in growth and immune function [255]. Zinc is the second most important micronutrient deficiency in infants after Iron [256]. Zinc deficiency has been reported to affect the growth of infants, especially those born preterm or small for gestational age [257].

Six studies addressed postnatal zinc supplementation in infants who were small at birth, but four of these apparently excluded preterm newborns and were not included in the present review [258-261].

One study [262] looked at postnatal zinc supplementation in very low birth weight infants (<1500 g), most of whom are likely preterm. There was a positive effect of supplementation on length gain, but not for weight or head circumference. There was also a positive effect on locomotor development.

A second study [263] assessed zinc supplementation in low birth weight infants (<2500 g) in a community based randomized controlled trial. The authors found that postnatal zinc supplementation is effective in reducing incidence of diarrheal episodes and helps in linear growth and weight gain in infants born with low birth weight.

##### Assessment

Notwithstanding the interesting findings in these studies, the overall quality of the evidence is low regarding the benefit of zinc supplementation in preterm infants, and there is a weak recommendation against the intervention until further studies with adequate samples and gestational age assessment are available.

#### Postnatal selenium supplementation

Selenium is involved in the human immune system and its deficiency can lead to impaired immunity [264,265].

A Cochrane review comprising three RCTs, [266] assessed the effect of postnatal selenium supplementation on mortality and morbidity in preterm infants. Selenium was associated with a reduction in one or more episodes of sepsis after seven days [RR= 0.73 (0.57, 0.93)]. However, no significant difference was seen in rates of mortality or oxygen use. There was a nonsignificant reduction in retinopathy of prematurity (any grade) among supplemented infants. The review concluded that postnatal selenium supplementation potentially reduced episodes of late onset neonatal sepsis but did not improve survival rates.

##### Assessment

While some experts have recommended selenium supplementation of preterm formulas (Klein 2002), the quality of the evidence is moderate due to the small number of studies, and there is a weak recommendation in favor of supplementation. Further studies are urgently needed.

#### Chlorhexidine treatment on the cord

Of the 4 million annual neonatal deaths, approximately 36% are attributable to infections [267] with the cord as a common portal of entry for organisms. The use of topical cord antiseptics has been proposed as a potential preventive measure [268]. Chlorhexidine appears to be particularly useful because of its wide-ranging activity against Gram-positive and Gram-negative bacteria [269], and the fact that it can be applied to the umbilical stump of the newborn as well as used for skin wiping and washing.

We identified two RCTs on the use of chlorhexidine on the umbilical stump. The first was restricted to preterm infants with gestational age <34 weeks and birth weight <2500g [270], while the other included both term and preterm infants [271]. The Pezzati trial compared chlorhexidine to salicylic sugar powder for umbilical cord care in preterm infants in neonatal care units, showing that the incidence of bacterial colonization was lower with salicylic group compared to chlorhexidine. There were no deaths in this trial, nor was there a control group with traditional cord care.

In Nepal, a community based trial showed that chlorhexidine use was associated with a 24% nonsignificant reduction (95% CI 0.55-1.04) in neonatal mortality compared to dry cord-care; among infants enrolled within the first 24 hours, mortality was significantly lower in the intervention group (RR 0.66; 95% CI 0.46-0.95) [271].

Chlorhexidine can also be used to cleanse the skin of the newborn. A recent large community-based RCT trial in rural Nepal [272] included 17,000 term and preterm infants. The intervention did not significantly affect mortality rates in term infants. However, a statistically significant reduction (28%) in mortality rates among low birth weight infants was documented (RR= 0.72, 95% CI= 0.55-0.95).

##### Assessment

Despite the interesting findings from these studies, the overall evidence for the benefits of chlorhexidine use among preterm infants is moderate and further trials are needed to validate impact before recommending this intervention for widespread use.

#### Community case management of sepsis and pneumonia in preterm neonates

The World Health Organization recommends parenteral antibiotic therapy in a health facility as the standard treatment for serious neonatal infections (i.e., septicemia, pneumonia, and meningitis) in LMICs [273]. However, this may not be the most feasible approach in low-income countries and alternative strategies may be needed [193]. Community case-management with antibiotics of neonatal sepsis and pneumonia has shown promising results [274]. The present review has examined the effect of this approach on the survival of sick preterm neonates.

Early studies of community management of neonatal sepsis did not include a sufficient number of preterm newborns for separate analyses [275]. In a similar study Datta et al [276] undertook a prospective controlled study of the case management of ARI in low birth weight infants, but did not distinguish preterm infants. They found a 64% reduction in case fatality due to ARI in the intervention area as compared to the control population, as well as declines of 24% (95% CI 6%-35%) in the infant mortality rates and 58% in the pneumonia specific mortality rates.

The study of home-based newborn care by Bang et al. [233] conducted in rural India found that case fatality in preterm neonates declined by 69.5 % (from 33.3 to 10.2%, p<0.0001). Case fatality in preterm low birth weight neonates without sepsis who received only supportive care decreased from 28.2 to 11.5% (p<0.01), and among those with sepsis who received supportive care and antibiotics decreased from 61% to 13.2% (p<0.005).

##### Assessment

Available data on community-based case management of neonatal sepsis and pneumonia in LMICs make a compelling case for providing such care in circumstances where referral may not be possible. While the overall evidence is moderate due to the small number of studies reporting on results among preterm infants, additional evidence is provided by studies including newborns in general, whether term or preterm. There is a strong recommendation in favor of this intervention in appropriate settings.

#### Kangaroo mother care (KMC)

The kangaroo mother care (KMC) method was developed in the 1970's in Colombia in response to overcrowded neonatal care units [277]. This method includes three main components: 1) skin-to-skin contact—a newborn baby is kept in a prone position between the mother's breasts several hours a day; 2) exclusive on-demand breastfeeding; and 3) early hospital discharge with appropriate follow-up. KMC is aimed at low birth weight babies, usually born preterm. It is applied after these babies are stabilized in terms of temperature, respiratory function and feeding [278]. The method has been recently adapted to non-hospital settings [279].

A systematic review carried out in 2003 [278] was updated to incorporate recent trials [279-281]. Four RCTs associated KMC with a reduction in severe morbidity (pooled RR 0.51; 95% CI 0.37-0.70). Five hospital-based studies reporting on neonatal mortality of infants under 2000 g, showed a pooled RR of 0.64 (95% CI 0.43-0.94). A community-based RCT in Bangladesh showed a significant reduction in neonatal mortality (RR 0.37; 95% CI 0.16-0.86) when analyses were restricted to newborns weighing 2000 g or less, but the difference in infant mortality was not quite significant (RR 0.56; 95% CI 0.30-1.05). It should be noted that 40% of the infants in this trial did not have a birth weight measure, so this latter analyses should be interpreted with caution. All studies are from LMICs.

##### Assessment

There is a high level of evidence that hospital-based KMC is an effective method of preventing severe morbidity and mortality, especially in low- and middle-income countries. The intervention is inexpensive and feasible, and therefore strongly recommended for hospital settings.

On the other hand, the evidence on community-based KMC is restricted to a single study with less clear results. In addition, that study failed to reach compliance with the recommended practice, which raises issues about its feasibility for widespread implementation [279]. New research is needed on the best ways to scale up the intervention at a community level and for estimating its impact on morbidity and mortality.

#### Early breastfeeding

Breastfeeding has many beneficial effects, including protection against neonatal morbidity and mortality, [282-284] and long-term improvements in cognitive function [285,286].

The composition of breast milk changes during the first week of life. Colostrum intake is particularly important due to its many antimicrobial properties [287]. Early breastfeeding ensures the infant's intake of colostrum is maximized, and also promotes the establishment of lactation by stimulating prolactin production.

The timely initiation of breastfeeding in the first hour of life is promoted by international organizations, such as UNICEF and WHO [288]. For preterm newborns, most of whom are unable to suckle until 34 weeks of gestational age or later, [284,289,290] early breastfeeding entails using a cup and spoon to administer expressed milk.

There is ample evidence from RCTs that early skin-to- skin contact, as in kangaroo mother care, increases the duration of total and exclusive breastfeeding [278,280,291,292]. Because early breastfeeding is associated with exclusive and total breastfeeding duration, and the latter with reduced morbidity and mortality, it is reasonable to expect that early breastfeeding will reduce these poor outcomes. However, only two observational studies conducted in Ghana and Nepal evaluated the effect of early breastfeeding on neonatal mortality, both showing that delayed initiation was associated with higher neonatal mortality [293,294]. A third study from Egypt also reported increased diarrhea incidence in the first six months of life among babies who were put to the breast three or more days after birth [295].

The literature comparing breast milk against formula feeding in early life is more abundant than that comparing early versus late breastfeeding initiation. A recent systematic review [284] found two RCTs and three cohort studies, all showing significant protective effects of human milk on systemic infections and necrotizing enterocolitis. A separate 2003 meta-analysis of four RCTs showed preterm babies receiving human milk were less likely to develop necrotizing enterocolitis (RR 0.34; 95% CI 0.12-0.99) than infants who received formula milk [283]. When a more recent study was added to this metaanalysis, [296] the pooled RR was equal to 0.28 (95% CI 0.11-0.69).

##### Assessment

There is a high quality of evidence that breast milk has beneficial effects in the prevention of necrotizing enterocolitis and severe infections in preterm babies. On the other hand, the level of evidence of an association between early breastfeeding and neonatal mortality is only moderate. The intervention is strongly recommended to be scaled-up due to its multiple benefits.

#### Thermal care immediately after birth

The normal body temperature of a newborn baby is between 36.5 and 37.5°C, and hypothermia refers to body temperature below 36.5°C [297]. Hypothermia is a danger sign in preterm babies, [298] and it has long been known that preventing heat losses in these babies improves survival [299]. Preterm babies are highly susceptible to hypothermia due to their low body fat, and this condition is highly prevalent in low- income countries [300-302].

A "warm chain" to prevent hypothermia in the newborn child includes 10 steps: 1) warm delivery room; 2) immediate drying; 3) skin-to-skin contact; 4) breastfeeding; 5) bathing and weighing postponed; 6) appropriate clothing/bedding; 7) mother and baby together; 8) warm transportation; 9) warm resuscitation; and 10) training and awareness raising [297]. Other methods to keep the baby's temperature include radiant heaters, polyurethane bags, cling films, thermal mattresses, incubators, and topical application of oil or paraffin-based ointments [303,304].

Because RCTs of thermal care versus no thermal care would not be ethical, available studies compare different methods. A Cochrane review [305] concluded use of plastic wraps or bags, transwarmer mattresses, and early skin-to-skin contact, all kept preterm and/or low birth weight babies warmer compared to routine thermal care. Another Cochrane review compared incubators with radiant heaters in preterm babies [306], showing no differences in severe infections or deaths, although radiant heaters were associated with greater water loss.

Two of these interventions—use of plastic wraps and early skin-to-skin contact—are particularly relevant for LMICs, and were subjected to new meta-analyses. Three studies of skin-to-skin contact [280,307,308] in low birth weight children were located, and the pooled RR of hypothermia was significantly lower in infants in the skin-to-skin group compared to those receiving routine care (0.13; 0.07-0.24). Three trials [309-311] and one observational study [312] assessed mortality impact in preterm babies, all with small sample sizes. The overall RR was 0.62 (95% CI 0.35-1.10). These four studies showed significant improvements in thermal control, as did another Indian trial for which mortality was not reported [313].

In addition, two recent community trials of essential newborn care packages showed hypothermia control can be feasibly scaled-up for home deliveries [314]; [315]. One of these compared a package including traditional skin-to-skin thermal care with a package including ThermoSpot, which is a liquid crystal sticker that indicates hypothermia by changing color. Hypothermia recognition and thermal care increased in both groups, but the ThermoSpot package did not show an advantage over the package of essential newborn care.

Three of the seven studies included in the two meta-analyses were from LMICs.

##### Assessment

The quality of the evidence supporting thermal control relative to absence of control is based on observational studies carried out in the distant past, and according to the GRADE guidelines is judged as moderate - even though RCTs would be unethical. Several different approaches were shown to provide adequate thermal control in RCTs. Of particular relevance to LMICs are skin-to-skin contact and plastic wraps, both of which are superior to routine thermal care in terms of keeping neonates warm. These provide high quality evidence. Comparisons of skin-to-skin contact relative to routine care suggest a possible impact on mortality, but the confidence interval is wide. The overall recommendation for skin-to-skin contact and plastic wraps is strong.

#### Application of continuous distending pressure to the lung for respiratory distress syndrome (RDS)

Continuous distending pressure (CDP) to the lung includes applying positive pressure via the nose or trachea or negative pressure around the chest, and is used to treat respiratory distress syndrome (RDS). In the absence of surfactant replacement therapy, CDP to the lung reduces the need for intubation and mechanical ventilation (pooled RR 0.72; 95% CI 0.56-0.91) and decreases the composite outcome of death or respiratory failure (pooled RR 0.65; 95% CI 0.52-0.81) [316]. However, CDP may increase the risk of pneumothorax (pooled RR 2.64; 95% CI 1.39-5.04). Compared to late administration, early CDP results in a lower use of intubation and mechanical ventilation (RR 0.55; 95% CI 0.32-0.96) [317].

Nasal CPAP refers to the application of CDP to the lungs via prongs placed in the nose and is a valid alternative to routine intubation and mechanical ventilation in treating RDS [318-324]. In a systematic review comparing prophylactic nCPAP to standard therapy among very preterm infants (<32 weeks or <1500 g) that included two studies, no significant differences in rates of intubation, bronchopulmonary dysplasia, mortality, pneumothoraces or other morbidities were noted [325].

Nasal CPAP may be particularly useful in low-resource settings. In Fiji, a pre-evaluation and post-evaluation showed a 50% reduction in the need for mechanical ventilation among newborns with RDS after introduction of nCPAP [326]. A small non-randomized study in South Africa found nCPAP reduced mortality by 50% compared with oxygen alone for preterm newborns between 26 to 28 weeks gestation with RDS [327].

Even in the surfactant era, a multicenter trial of 610 infants between 25 and 28 weeks gestation randomized to either nCPAP or intubation and mechanical ventilation within 5 minutes of birth found no differences in the primary outcome of BPD or death [328], but fewer infants who received CPAP required oxygen at 28 days after birth. Pneumothorax was more frequent in the nCPAP group perhaps because they did not receive early surfactant.

Nasal CPAP is best delivered using bi-nasal prongs while the end expiratory pressure is generated by placing the expiratory limb of a constant flow circuit into water at a depth sufficient to generate the required pressure (bubble CPAP) [329-331]. Other methods are more expensive and may not be more effective [331-333].

##### Assessment

The quality of evidence of nCPAP for treatment of respiratory failure among preterm newborns to prevent death, bronchopulmonary dysplasia, or both is high and this modality is used routinely in developed countries. In addition, the quality of evidence for the use of nCPAP as an alternative to mechanical ventilation is high, making it particularly appealing in LMIC settings even though only two of the 29 studies reviewed are from such settings. The intervention is strongly recommended, however optimal delivery systems, interfaces and optimal pressures must be elucidated. Further, ensuring adequate support services such as managing airleak syndrome will be important prior to widespread implementation.

#### Intravenous immune globulin (IVIG)

Preterm neonates are deficient in immunoglobulins, particularly IgG, [334] and the use of non-specific, polyclonal intravenous immune globulin (IVIG) (normal human IgG immunoglobulin) has been advocated to prevent or treat neonatal infections.

A Cochrane meta-analysis reviewed 19 studies from different parts of the world that compared IVIG with placebo [335] in approximately five thousand preterm or low birth weight newborns. The use of prophylactic IVIG reduced sepsis (RR 0.85; 95% CI 0.74-0.98), but did not significantly reduce all-cause mortality (RR 0.89; 0.751.05) or mortality due to infectious diseases (RR 0.83; 95% CI 0.56-1.22). The same authors also reviewed nine RCTs restricted to neonates who had a suspected infection during their initial hospital stay [336]. Based on six trials, there was a borderline reduction in overall mortality in the IVIG group (RR 0.63; 95% CI 0.40-1.00). Seven trials of newborns with confirmed infection showed IVIG did result in a statistically significant reduction in mortality (RR 0.55; 95% CI 0.31-0.98). However, the authors identified methodological problems, such as lack of allocation concealment, lack of blinding in outcome assessment, high frequency of post-randomization exclusions and lack of long-term follow-up, and suggested further trials be conducted to confirm or refute the effectiveness of IVIG treatment. [336] More recent trials on this subject are not available.

##### Assessment

The level of evidence is high, but the intervention has a weak recommendation in favor of adoption. More information on dosing, potential risks[337] and long-term follow-up is needed before IVIG may be widely recommended. The following issues are of particular relevance to LMICs: feasibility (e.g., the need for cold storage and for intravenous administration); cost-benefit analyses; and ensuring formulations include standardization of proper levels of antibodies against prevalent infectious agents.

#### Surfactant replacement therapy for respiratory distress syndrome (RDS)

A single dose of bovine, calf, human or synthetic surfactant to prevent RDS is associated with a decrease in neonatal mortality and chronic lung disease, or bronchopulmonary dysplasia (BPD) [338,339]. Prophylactic use of surfactant is more effective than administration after RDS symptoms develop, but it is substantially more costly and benefits are limited to very preterm newborns (<30 weeks gestation) [340].

Rescue surfactant treatment provided within two hours of delivery, compared with later administration, is associated with decreases in neonatal mortality, chronic lung disease and chronic lung disease or death at 36 weeks [341]. Three to four doses of surfactant for preterm neonates appear to be more beneficial than single doses for prevention or treatment of RDS with respect to incidence of pneumothorax; however, differences in mortality were not statistically significant in this recent meta-analysis [342]. These findings are of particular relevance to LMICs, as treatment with surfactant within the first two hours of life may reduce mortality while requiring treatment of a substantially smaller number of neonates than the prophylaxis approach. Antenatal steroids are shown to be synergistic with surfactant replacement [343]. Therefore, it is possible that with optimal antenatal steroid coverage fewer neonates would require intubation and those receiving surfactant therapy may further improve their outcomes.

Natural surfactants, compared to synthetics, result in reduced mortality and pneumothorax [344]. Clinically significant differences between porcine and bovine surfactants have not been consistently demonstrated [345,346]. However, comparisons of natural surfactants to new synthetic surfactant preparations, which contain surfactant protein analogues, found no differences in mortality at 36 weeks, chronic lung disease, the combined outcome of mortality or chronic lung disease, or mortality at one year [347,348]. Synthetic forms of surfactant exhibit more temperature stability and have the potential for scaling up production to lower costs. These features are particularly relevant in LMICs.

Surfactant is administered to the newborn lung after endotracheal intubation, which is associated with significant cost and potential morbidity. Alternative surfactant delivery systems have the potential to greatly improve access to and safety of this intervention in both LMICs and HICs. Attempts at nebulizing surfactants have been unsuccessful [345,349]. More recently, surfactant instilled into the posterior pharynx is shown to be feasible without intubation and to improve lung function, yet further research is needed to assess the efficacy of such an approach [350-352].

##### Assessment

The quality of evidence for using surfactant replacement therapy to prevent or treat respiratory failure, bronchopulmonary dysplasia and/or death among preterm newborns is high and this modality is used routinely in HICs. Although only three of the 50 studies reviewed are from LMICs, the intervention is strongly recommended. However, multiple challenges remain and should be addressed prior to widespread implementation: identification of optimal dosing and delivery systems, development of low-cost preparations, and refined techniques to identify high-risk newborns who may benefit from surfactant therapy.

### Community-based packages of interventions

So far, this review was centered on standalone biological, behavioral or health care interventions. Community-based strategies in LMICs, however, often combine several different interventions, making it impossible to disentangle their individual effects. Here we review studies of community-based intervention packages that provide maternal and newborn care.

Nearly half of the world's 124 million births that take place every year occur at home. In South Asia and sub-Saharan Africa—the two regions with the highest neonatal mortality rates in the world—64% of the births take place at home. Most of these births are without skilled attendance. In these areas, the most promising short-term strategy for providing newborn care entails training and outfitting community health workers. Bhutta and colleagues conducted a thorough review of the literature on community-based interventions aimed at reducing perinatal and neonatal morbidity and mortality [353]. All six trials included in this review were from South Asia and had a significant impact on newborn health. Similar studies were not available from other regions of the world.

The classic studies of Bang and associates in Gadchiroli, India, showed an important impact of home-based care by trained village health workers, who provided antenatal care, managed birth asphyxia, hypothermia, neonatal sepsis and secured the care of preterm and low birth weight newborns. This low-cost, sustainable intervention reduced case-fatality of neonatal sepsis, leading to a 71% reduction in perinatal mortality and a 62% reduction in neonatal mortality rates [274,354-356].

In Nepal, a RCT evaluated the impact of a participatory intervention involving women's groups convened by a female facilitator. During these sessions, local problems were identified and possible solutions proposed. Women in the intervention clusters had more antenatal care, institutional deliveries and, skilled birth attendance, and hygienic care than those in the control areas. These changes were accompanied by significant reductions in neonatal and maternal mortality in the study areas (adjusted odds ratios 0.70, 95% C.I. 0.53-0.94 and 0.22, 95% C.I. 0.05-0.90, respectively) [357].

In Sind, Pakistan, a RCT was conducted in which traditional birth attendants were trained and received disposable delivery kits, and female health workers made the connection of traditional services with health institutions. Women in the intervention communities had more antenatal care and were delivered more often with safe kits. The odds ratio for perinatal mortality in the intervention areas was 0.70 (0.59-0.82) [142].

In a pilot randomized study in Pakistan, female health workers and traditional birth attendants received training in a package of community-based intervention for improving perinatal care. The proportion of deliveries by skilled heath attendants increased in the intervention villages and there were significant reductions in the rates of stillbirth and neonatal death [140].

Another recent RCT conducted in Bangladesh compared three groups: a home-care program (consisting of antenatal and post-natal home visits by community health workers who provided care directly to mothers and newborns), a community-care program (including group sessions on birth and newborn-care preparedness and promotion of careseeking from qualified providers) and a comparison group. In the last 6 months of the 30 month intervention, the neonatal mortality was reduced by 34% in the home-care program (adjusted relative risk 0.66, 95% C.I. 0.47-0.93) relative to the comparison group [314]. No impact was observed in the community-care group.

Finally, a RCT conducted in Uttar Pradesh, India, entailed the comparison of three groups. The control group received the usual maternal and newborn services; the first intervention group received a preventive package of essential newborn care interventions, including birth preparedness, clean delivery and cord care, thermal care (with skin-to-skin contact), breastfeeding promotion, and training on recognition of danger signs; the second intervention group received the same basic package described above plus the use of a liquid crystal hypothermia indicator. Both control groups showed improvements in birth preparedness, hygienic delivery, thermal care, umbilical cord care, skin care, and breastfeeding. In comparison with the control group, neonatal mortality was reduced by 54% in the essential newborn care group (rate ratio 0.46, 95% C.I. 0.35-0.60) and by 52% in the essential newborn care plus ThermoSpot group (rate ratio 0.48, 95% C.I. 0.35-0.66) [315].

#### Assessment

Taken together, these six trials in South Asian countries strongly suggest that community-based programs, particularly those that provide maternal and newborn care by trained village health workers, may have a substantial impact in reducing stillbirth and neonatal mortality. The quality of the evidence is high, and there is a strong recommendation for their implementation in settings where the vast majority of deliveries occur at home.

None of these trials reported impact on preterm deliveries. Also, trials from other regions of the world— particularly sub-Saharan Africa—are urgently needed.

### Research questions: development of interventions for preterm birth and stillbirth

The review of the literature, summarized above, led to the identification of over 100 research questions. These questions were analyzed and prioritized by workgroups at the International Conference on Prematurity and Stillbirth and will be addressed in a forthcoming Child Health and Nutrition Research Initiative (CHNRI) paper. Workgroup participants are leading international experts in the areas of maternal, fetal newborn and child health.

## Conclusion

After reviewing 2,000 studies on more than 80 interventions, 21 preterm birth and stillbirth interventions are strongly recommended in LMICs. Each of these has high quality evidence it is effective in low-income settings. This extensive review identifies eight proven effective interventions for preventing stillbirth in LMICs and 11 for improving the survival of the preterm neonate. Only two available interventions are strongly recommended for preventing preterm birth.

Additional research must be conducted on many other promising interventions before they can be recommended for scale-up in LMICs. Additional research on the cost-effectiveness of these interventions is also needed, as it was outside the scope of this analysis. It must also be noted that many interventions not strongly recommended for these specific outcomes are indeed recommended for other related maternal and child health outcomes. A summary of these findings, based on the GRADE assessments, is provided in Table 3. The next article discusses scale-up of proven interventions, and addresses delivery in the context of maternal, newborn and child health [3].

## Authors' contributions

FB wrote sections relating to prevention of preterm birth, ZB wrote sections on stillbirth, and MB wrote sections on improving survival of preterm newborns. CV contributed to the review and coordination of all sections. TH contributed to the section on preterm newborn interventions. CR helped draft and conceive of this article as part of a global report on preterm birth and stillbirth, and participated in its design, coordination, and review. The article was reviewed by all authors.

## Competing interests

The authors declare that they have no competing interests.
